# Pharmacological and molecular dynamics analyses of differences in inhibitor binding to human and nematode PDE4: Implications for management of parasitic nematodes

**DOI:** 10.1371/journal.pone.0214554

**Published:** 2019-03-27

**Authors:** Kevin D. Schuster, Mohammadjavad Mohammadi, Karyn B. Cahill, Suzanne L. Matte, Alexis D. Maillet, Harish Vashisth, Rick H. Cote

**Affiliations:** 1 Department of Molecular, Cellular, and Biomedical Sciences, University of New Hampshire, Durham, NH, United States of America; 2 Department of Chemical Engineering, University of New Hampshire, Durham, NH, United States of America; Carl von Ossietzky Universitat Oldenburg, GERMANY

## Abstract

Novel chemical controls are needed that selectively target human, animal, and plant parasitic nematodes with reduced adverse effects on the host or the environment. We hypothesize that the phosphodiesterase (PDE) enzyme family represents a potential target for development of novel nematicides and anthelmintics. To test this, we identified six PDE families present in the nematode phylum that are orthologous to six of the eleven human PDE families. We characterized the binding interactions of family-selective PDE inhibitors with human and *C*. *elegans* PDE4 in conjunction with molecular dynamics (MD) simulations to evaluate differences in binding interactions of these inhibitors within the PDE4 catalytic domain. We observed that roflumilast (human PDE4-selective inhibitor) and zardaverine (selective for human PDE3 and PDE4) were 159- and 77-fold less potent, respectively, in inhibiting *C*. *elegans* PDE4. The pan-specific PDE inhibitor isobutyl methyl xanthine (IBMX) had similar affinity for nematode and human PDE4. Of 32 residues within 5 Å of the ligand binding site, five revealed significant differences in non-bonded interaction energies (van der Waals and electrostatic interaction energies) that could account for the differential binding affinities of roflumilast and zardaverine. One site (Phe506 in the human PDE4D3 amino acid sequence corresponding to Tyr253 in *C*. *elegans* PDE4) is predicted to alter the binding conformation of roflumilast and zardaverine (but not IBMX) into a less energetically favorable state for the nematode enzyme. The pharmacological differences in sensitivity to PDE4 inhibitors in conjunction with differences in the amino acids comprising the inhibitor binding sites of human and *C*. *elegans* PDE4 catalytic domains together support the feasibility of designing the next generation of anthelmintics/nematicides that could selectively bind to nematode PDEs.

## Introduction

The efficacy of—and resistance to—anthelmintic/nematicidal compounds for controlling parasitic nematodes is a growing concern in the fields of medicine, veterinary medicine, and agriculture. Widespread administration of current drugs to treat human diseases in impoverished regions of the world may result in increasing levels of resistance in human parasitic nematodes [1]. Similarly, reduced livestock health and profitability as a result of anthelmintic resistance poses growing challenges to this industry [2]. In the United States, plant-parasitic nematodes account for an estimated 8–15% of all crop losses and cause approximately $100 billion in annual damages [3–5]; the historical use of organophosphates or carbamates have been greatly restricted due to their health and environmental hazards [6, 7]. Hence, there is an urgent need for the development of novel compounds to address the growing resistance to anthelmintics and the toxicity of most chemical nematicides. An ideal anthelmintic/nematicide would disrupt the parasitic nematode lifecycle while leaving the host and other organisms unaffected.

The phylum Nematoda is diverse and has been historically categorized into five main clades [8]. Information regarding the genomics and physiology of parasitic nematodes is limited, especially in comparison to the free-living nematode, *C*. *elegans*, whose genome and nervous system has been fully mapped and has served as a model organism for studying nematode development and behavior [9].

Cyclic nucleotide metabolism is of central importance for a wide range of physiological processes in nematodes, as attested by the presence in *C*. *elegans* of 38 genes that synthesize cAMP or cGMP [10], as well as six phosphodiesterase (PDE) genes [11, 12]. The cAMP second messenger has been linked to various behaviors including feeding and locomotion [13, 14]. Neural pathways responsible for sensory signaling (chemoreceptors, thermotaxis, phototaxis) appear to be regulated through cGMP signaling pathways [11, 15–17]. Furthermore, RNAi, gene deletion, and pharmacological studies have shown that altered PDE activity can cause lethality, sterility, aberrant locomotion, lethargy, and altered development in *C*. *elegans* [18–22]. Some of the observed phenotypes (e.g., lethality, sterility) are clearly relevant to developing effective anthelmintics/nematicides that specifically target parasitic nematodes—but not other animal phyla. Indeed, PDEs and their inhibitors are being investigated for their therapeutic potential in combatting various protozoal diseases [23]. Another advantage to targeting phytoparasitic nematode PDEs for the development of nematicides is the greatly reduced likelihood that a PDE inhibitor-based nematicide would have adverse effects on plants, since Class I PDEs have not been identified to date in plants [24].

The Class I PDE superfamily in vertebrates consists of eleven PDE families that have been identified throughout the animal kingdom [25]. The eleven families are distinguished by differences in their substrate specificity, modes of regulation, pharmacological properties, and tissue distribution [26]. However, all Class I PDE enzyme families share a conserved Prosite domain signature (Prosite PS00126; https://prosite.expasy.org/PDOC00116) in the catalytic domain consisting of the amino acid sequence pattern HD[LIVMFY]xHx[AG]xx[NQ]x[LIVMFY]. The crystal structures of the catalytic domains of almost all the PDE families have been solved, providing atomic-level details on the enzymatic and pharmacological properties of this enzyme superfamily [27]. The catalytic domains of the Class I PDE superfamily are made up of ~330 amino acids whose secondary structure consists of 16 α-helices. These α-helices create three subdomains [28] which form a deep catalytic pocket at their center. The active site is composed of two sub-pockets, which bind two divalent metal ions and the substrate, respectively [27]. Zinc and magnesium ions are stabilized by conserved His and Asp residues in the metal binding pocket [27]. The crystal structure of human PDE4 catalytic domain in a complex with 5’-AMP [29] has revealed that cyclic nucleotides are stabilized by ionic interactions with the bound divalent cations and with Asp and His residues in the metal binding pocket, as well by hydrophobic interactions with conserved Gln and Phe residues in the hydrophobic pocket. The invariant Gln residue of PDEs have been shown to be critical for substrate and inhibitor binding [27].

The extensive literature on human PDE inhibitor pharmacology [30] and the commercial availability of many types of family-specific PDE inhibitor compounds enabled us to experimentally evaluate the potential of PDE inhibitors to serve as chemical nematicides targeting parasitic nematodes. In this paper, we present work that supports our hypothesis that PDEs in parasitic nematodes represent a viable target for anthelmintic or nematicidal compounds. We have identified the PDE families present in the nematode phylum and show that nematode PDEs are evolutionarily divergent from PDEs in other animal phyla. We have subcloned and expressed the catalytic domain of *C*. *elegans* PDE4 to demonstrate that compounds designed to selectively inhibit human PDE4 were less potent in inhibiting nematode PDE4; this result supports the notion that compounds can be identified in the future that selectively target nematode PDEs over human PDEs. Finally, we have used atomistic molecular dynamics simulations (an approach used previously to compare the binding of inhibitors to different human PDE4 isoforms [31, 32]) to investigate the role of structural differences in inhibitor interactions in human and nematode PDE4 that underlie the different pharmacological properties of nematode and human PDE4. Together, these results support the idea that differences in the inhibitor binding site of nematode PDEs can be exploited to rationally design nematode-selective PDE inhibitors that act as an anthelmintic or nematicide without adverse effects on vertebrate animals or crops.

## Materials and methods

### Identification and phylogenetic analysis of PDEs

The sequences for the eleven phosphodiesterase families in humans (21 genes) were obtained from UniProt (www.uniprot.org). The protein sequences for the six PDE families identified in *C*. *elegans* were retrieved from Wormbase (www.wormbase.org [9]). The *C*. *elegans* sequences listed in Wormbase consist of multiple isoforms that do not differ in sequence within the catalytic domain, hence we selected the longest isoform for analysis.

The phylogenomic pipeline we used is based on publicly available, whole-genome data from the following organisms that are representative of different animal phyla: chordates (*Danio rerio*, *Ciona savignyi*, *Branchiostoma belcheri*); arthropods (*Drosophila melanogaster*, *Daphnia pulex*, *Ixodes scapularis*); hemichordates (*Saccoglossus kowalevskii)*; and annelids (*Capitella telata*). We also included the following nematode species: *Caenorhabditis elegans* (free-living), *Pristionchus pacificus* (free-living), *Brugia malayi* (human parasite), *Strongyloides ratti* (human parasite), *Onchocerca volvulus* (human parasite), as well as the following plant-parasitic nematode species: *Globodera rosochiensis*, *Globodera pallida*, *Meloidogyne floridensis*, *Meloidogyne hapla*, *Meloidogyne incognita*, and *Bursaphelenchus xylophilus*. The nematodes included in this analysis are representative of nematode Clades III, IV, and V [8]. The nematode genomic databases were obtained from Wormbase [9]. Database information can be found in S1 Table.

The full length sequences of the 21 human and 6 *C*. *elegans* PDEs were used as Basic Local Alignment Search Tool [BLAST; [33]] queries of the collected databases of the species listed above. The top 50 genes that met a low stringency requirement threshold of 10^−2^ were kept. These data were then compiled into a single file and redundant sequences were removed using cdhit (http://weizhongli-lab.org/cd-hit/ [34]).

All the compiled sequences were then aligned using MAFFT (https://mafft.cbrc.jp/alignment/software/ [35]) and any gaps that had less than 20% occupancy were removed using trimAL (http://trimal.cgenomics.org/ [36]). To ensure we only included likely PDE sequences we removed sequences that had fewer than 300 total amino acid residues. We also eliminated any sequences that contained less than 200 residues within the catalytic domain [as defined by the *H*. *sapiens* PDE4 crystal structure (PDB ID: 3G4L; [37]]. Finally, we eliminated Daphnia_7212, Homo_31687, Danio_2743, Homo_101687, Meloidogynefloridensis_25761, and Meloidogyneincognita_3488, since they were missing one or more amino acid residues that make up the PDEase_I domain signature (Prosite PS00126) described above. The sequences obtained from the phylogenomic pipeline and those that were removed for the above-mentioned reasons are listed in S2 Table.

Phylogenetic trees were then created using RAxML 8.0 (https://cme.h-its.org/exelixis/web/software/raxml/ [38]) and the model with the highest support as determined by bootstrapping analysis was visualized using FigTree v1.4.3 (http://tree.bio.ed.ac.uk/software/figtree/). Bootstrapping support for the phylogenies were conducted with 100 bootstrap replicates.

### Expression and purification of *H*. *sapiens* and *C*. *elegans* PDE4

The pET15b vector containing the human PDE4D catalytic domain (residues 252–579, based on the numbering of the PDE4D3 isoform; NP_006194) was transformed into *E*. *coli* BL21 (DE3) cells for recombinant expression. Cells were grown to mid-exponential phase (OD_600_ ~0.6) and then incubated with 0.3 mM IPTG at 30°C with vigorous shaking for four h. The cells were harvested, the cell pellets resuspended in 30 mL of lysis buffer (20 mM Tris, 100 mM NaCl, pH 8.0) and centrifuged again for 10 min at 5000 RPM at 4°C. The resuspended pellet was incubated for 15 min at 4°C in lysis buffer supplemented with bacterial protease inhibitor cocktail (Sigma #P8849), 0.3 mM phenylmethylsulphonyl fluoride, and Lysonase Bioprocessing Reagent (Millipore-Sigma), and then lysed using a French press. Following centrifugation (23,000 x *g* for 20 min at 4°C), the supernatant was purified by passage through a nickel-nitrilotriacetic acid column and the His-tagged PDE4D protein was eluted with a linear concentration gradient of 0–300 mM imidazole.

The catalytic domain (amino acid residues 275–608) of *C*. *elegans* PDE4 isoform h (Uniprot ID: S6FCW6) was codon-optimized for bacterial expression and subcloned into the pMAL vector (containing the maltose binding protein fusion partner). *E*. *coli* BL21(DE3) cells containing this plasmid were grown to mid-exponential phase (OD_600_ ~0.6) and then induced using 1 mM IPTG for 20 h at 15°C. Following centrifugation, the washed cell pellet was resuspended in 50 mL of lysis buffer supplemented with 2 mM EDTA, 5 mM DTT, bacterial protease inhibitor cocktail, 1 mM phenylmethylsulphonyl fluoride, and Lysonase Bioprocessing Reagent. Cells were disrupted by sonication and centrifuged. The resulting supernatant was applied to an amylose-agarose column, washed, and then eluted with 10 mM maltose.

### Protein and enzyme activity assays

Protein concentrations were determined using the Bradford Assay [39], and the purity of the affinity-purified catalytic domains evaluated by SDS-PAGE. Hydrolysis of cyclic nucleotides was measured by a radiotracer assay [40]. Pharmacological studies of IBMX, zardaverine, and roflumilast (obtained from Millipore-Sigma or SelleckChem) were conducted in PDE assay buffer (20 mM Tris (pH 7.5), 10 mM MgCl_2_, and 0.1 mg/ml bovine serum albumin) containing 1 μM [^3^H]cAMP. The initial rate of cyclic nucleotide hydrolysis was determined by measuring cyclic nucleotide hydrolysis at three time points for each concentration of inhibitor tested. Dose-response relationships were analyzed using non-linear regression analysis (3-parameter logistic equation) with SigmaPlot (SPSS, Inc., Chicago, IL). IC_50_ values are reported as mean ± standard error of the mean (S.E.M.).

### Molecular dynamics simulations

The initial coordinates for protein structures were obtained from the crystallographic structures of PDE4D bound to ligands with PDB codes: 1ZKN [37], 3G4L [41], and 1MKD [42]; in each instance, only one structure was modeled, since the crystal structures of PDE4D consisted of identical PDE catalytic domains that co-crystallized into an oligomeric crystal. Numbering of amino acid residues for human PDE4D sequences used for MD simulations reflect the PDE4D3 amino acid sequence. For simulation studies of *C*. *elegans* PDE4 with bound inhibitors, we created structural models using SwissModel (https://swissmodel.expasy.org/ [43]) with each of the previously mentioned PDE4D crystal structures as templates. The amino acid numbering for the *C*. *elegans* PDE4 catalytic domain was arbitrarily numbered 1 (N285) through 324 (P608) (S3 Table) because no canonical sequence for *C*. *elegans* PDE4 is available to use as a reference for numbering purposes.

Upon comparing the *C*. *elegans* homology models with their templates, we observed that each model had initial mean-squared deviations (relative to their templates) of 0.12 Å, indicative of the homology models having a high structural similarity to the human PDE4 crystal structures. The stability of homology models was further tested using all-atom and explicit-solvent MD simulations. The stability of the *C*. *elegans* structural homology models in our MD simulations [in conjunction with the highly conserved nature of the PDE catalytic domain structure (PDEase_I; Pfam PF00233)] support their usefulness in the absence of experimentally determined structures.

We prepared six systems for MD simulations: three each for human PDE4D and *C*. *elegans* PDE4. Each system was then solvated with explicit TIP3P [44] water molecules, and charge-neutralized with counter-ions resulting in various system sizes (S4 Table). We used the software NAMD (http://www.ks.uiuc.edu/Research/namd/ [45]) for all MD simulations, and VMD (http://www.ks.uiuc.edu/Research/vmd/ [46]) for system setup and post-processing analysis.

CHARMM36 [32] force field was used including the CMAP correction [47, 48] for protein structures, and developed force-fields for all inhibitors using MATCH (https://brooks.chem.lsa.umich.edu/index.php?page=match&subdir=articles/resources/software [49]). We used periodic boundary conditions [50] and computed long range-electrostatics using the particle-mesh Ewald summation [51] with a grid spacing of 1 Å, an integration time-step of 2 fs, and a cutoff-distance of 10 Å for van der Waals interactions; these settings are typically used for conducting MD simulations of solvated systems of proteins using NAMD [45]. We first energy minimized each system, and continued production runs of each system in the NPT-ensemble for 120 ns using a Langevin thermostat and Nosé-Hoover barostat [52]. We also carried out an independent run with the same length of simulation for each system, giving two production runs for each prepared system. Additionally, we carried out simulations of the same length for the apo states of human PDE4D and *C*. *elegans* PDE4 (S4 Table).

### Nonbonding interaction energy calculations

To investigate the role of individual amino acids in the binding pocket of each protein/ligand complex, we computed non-bonded interaction energies between all atoms of ligands and those of residues forming the binding pocket (i.e., within 5 Å from each ligand). Interaction energy values were estimated by splitting them into electrostatic and van der Waals interactions, as follows:

$${\Delta E}_{non-bonded}={\Delta E}_{elec}+{\Delta E}_{vdW}$$

We carried out these calculations by including all frames in each MD trajectory.

### Dynamic cross-correlation analysis

The dynamic cross-correlation (DCC) maps of each system were calculated based on the C_α_ atoms of residues using the MD-TASK package (https://md-task.readthedocs.io/en/latest/home.html [53]). Each cell value (C_ij_) in the matrix of the DCC map were calculated using the following formula:

$$C_{ij}=\frac{\langle \Delta r_{i}.\Delta r_{j}\rangle }{\left(\sqrt{\langle {\Delta r_{i}}^{2}\rangle }.\sqrt{\langle {\Delta r_{j}}^{2}\rangle }\right)}$$

With Δr_i_ represents the displacement from the mean position of atom i, and < > denotes the time average over the whole trajectory. Positive values of C_ij_ show correlated motion between residues i and j, moving in the same direction, whereas negative values of C_ij_ show anti-correlated motion between residues i and j, moving in the opposite direction.

### Analysis of salt-bridging interactions

The salt-bridging interaction analysis was carried out using VMD based on a distance criterion uniformly applied to determine the existence of salt-bridges for each frame in all trajectories [54]. Specifically, the formation of a salt-bridging interaction was considered if the distance between any of the oxygen atoms of acidic residues and the nitrogen atoms of basic residues were within a cut-off distance of 3.2 Å.

## Results

### Identification of class I PDEs in the nematode phylum

To determine the PDE family members present in the nematode phylum, we performed BLAST searches of a representative set of protostome and deuterostome genomes (see *Materials and Methods* and S1 Table). As expected, our results identified the 11 vertebrate PDE families including each isoform previously identified in *H*. *sapiens* and *D*. *rerio* [25]. Non-nematode protostome genomes included in the analysis contain different sets of orthologs of the vertebrate PDE families (S1 Fig). Our BLAST results also confirmed the previously described six PDE families in *C*. *elegans* that were originally designated PDE-1, PDE-2, PDE-3, PDE-4, PDE-5, and PDE-6 [11, 12]. We found sequence data for the same six families in the human and animal parasitic nematode species (*S*. *ratti*, *B*. *malayi*, and *O*. *volvulus*) as well as in all but one of the plant-parasitic nematode species (*B*. *xylophilus*, *Globodera spp*., and *Meloidogyne spp*; see Table 1). Due to the draft formats of the genomic data of most parasitic nematode species, we used the data of multiple species within the same genus (when present) and treated them as the same organism for this analysis. The failure to identify a PDE2 ortholog in *P*. *pacificus* (Table 1) is likely due to the incomplete genome assembly currently available for this species.

**Table 1:** Identification of nematode PDEs from the phylogenomic pipeline.

Nematode Genus	PDE1	PDE2	PDE3	PDE4	PDE8	PDE10
*Caenorhabditis elegans* (Clade V)	NP_001129790.1 NP_493343.1	NP_001022705.2	NP_001293572.1 NP_001254452.1 NP_00125445.4 NP_001293571.1	NP_871945.1 NP_495601.1 NP_001040798.1 NP_871944.	NP_490787.1	NP_491544.3
*Pristionchus pacificus* (Clade V)	16690 16689		2021	2021	11647	PDM69049.1
*Strongyloides ratti* (Clade IV)	XP_024504037.1	XP_024507051.1	XP_024498853.1	XP_024509991.1	XP_024504870.1	XP_024505768.1
*Globodera pallida* (Clade IV)	8612 8613	676 14144 7739	510	11976 3887 1406	5161 9957	6470 7042
*Meloidogyne spp*. (Clade IV)	Hapla_9145 Floridensis_2805	Incognita_3487 Incognita_9561 Incognita_3488 Floridensis_46727 hapla_891	Floridensis_30127 Incognita_7615 Floridensis_25761 Hapla_13712	Hapla_4882 Hapla_4881 Floridensis_18998 Incognita_9738 Floridensis_20716	Hapla_14035 Floridensis_5969	Hapla_8166 Floridensis_49637 Floridensis_45771 Floridensis_13842 Hapla_8164
*Bursaphelenchus xylophilus* (Clade IV)	10439	5762	12230	15631	6750	2916
*Brugia malayi* (Clade III)	CTP81899.1	8657	XP_001896262.1	8819	CRZ23681.1	CTP81734.1
*Onchocerca volvulus* (Clade III)	7198	10576	947	370	3032	3346

Protein accession numbers in the table were used (when available) to replace the descriptors generated by the phylogenomic pipeline analysis. In some cases, multiple predicted PDE isoforms were found, but only one accession number is given when the catalytic domain amino acid sequence was identical. Protein databases used, the original accession numbers, and the labels assigned for the phylogenomic analyses are provided in S2 Table.

A multiple sequence alignment was then created using MAFFT, and RAxML was used to construct a phylogenetic tree (S1 Fig). We established *C*. *elegans* PDE-1, PDE-2, PDE-3, PDE-4, PDE-5, and PDE-6 as orthologs of vertebrate PDE1, PDE2, PDE3, PDE4, PDE10, and PDE8, respectively. For clarity, we will henceforth identify nematode PDE orthologs using the vertebrate PDE classification numbers (i.e., *C*. *elegans* PDE-5 will be referred to as PDE10).

**Fig 1:**
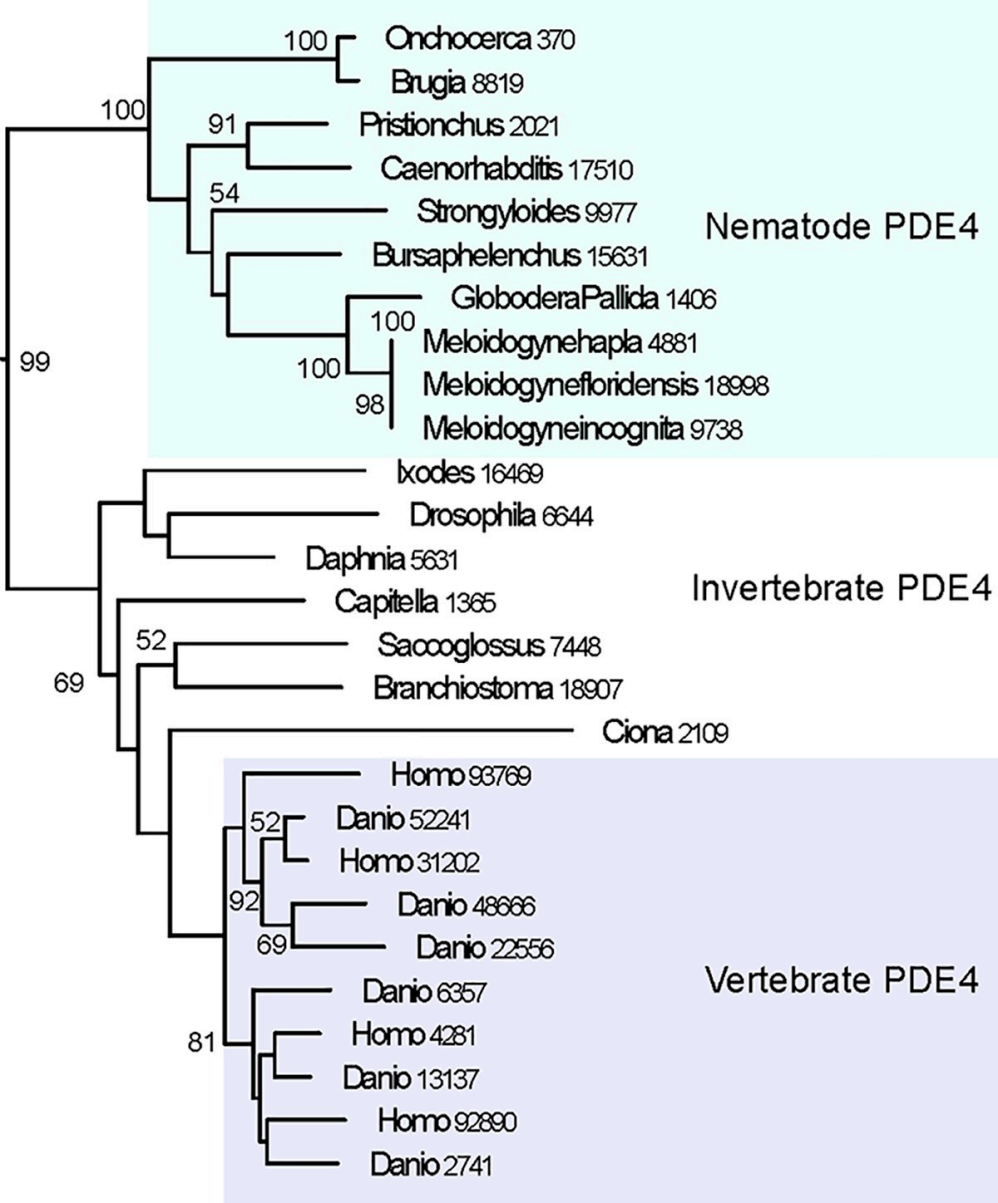
The PDE4 clade for the species involved in our analysis (*see Supporting Information, S1 Fig*). Bootstrap analysis was run 100 times and confidence values greater than 50 are displayed. Vertebrate PDE4s form a clade distinct from other phyla.

Based on our phylogeny, the nematode PDE1 and PDE10 clades are more closely related to other deuterostome species (*I*. *scapularis*, *D*. *melanogaster*, and *D*. *pulex*). For PDE10, nematodes are grouped with both protostomes and with *B*. *belcheri* and *S*. *kowalevskii* (both deuterostomes), while chordates, tunicates, and hemichordates form a distinct clade. For PDE1, there is a clearer distinction of nematodes grouping with arthropods (all protostomes). However, in the case of the nematode PDE2, PDE3, PDE4 (Fig 1), and PDE8 orthologs, the nematode PDE sequences are found in a clade distinct from the other protostomes and deuterostomes used in this analysis. As seen in Fig 1, nematode PDE4s all belong to the same clade, which is distinct from the groupings of vertebrate and non-nematode PDE4 sequences. Furthermore, whereas vertebrate genomes contain multiple genes for several of the PDE families, nematode genomes appear to only encode for a single gene for each nematode PDE family.

**Fig 2:**
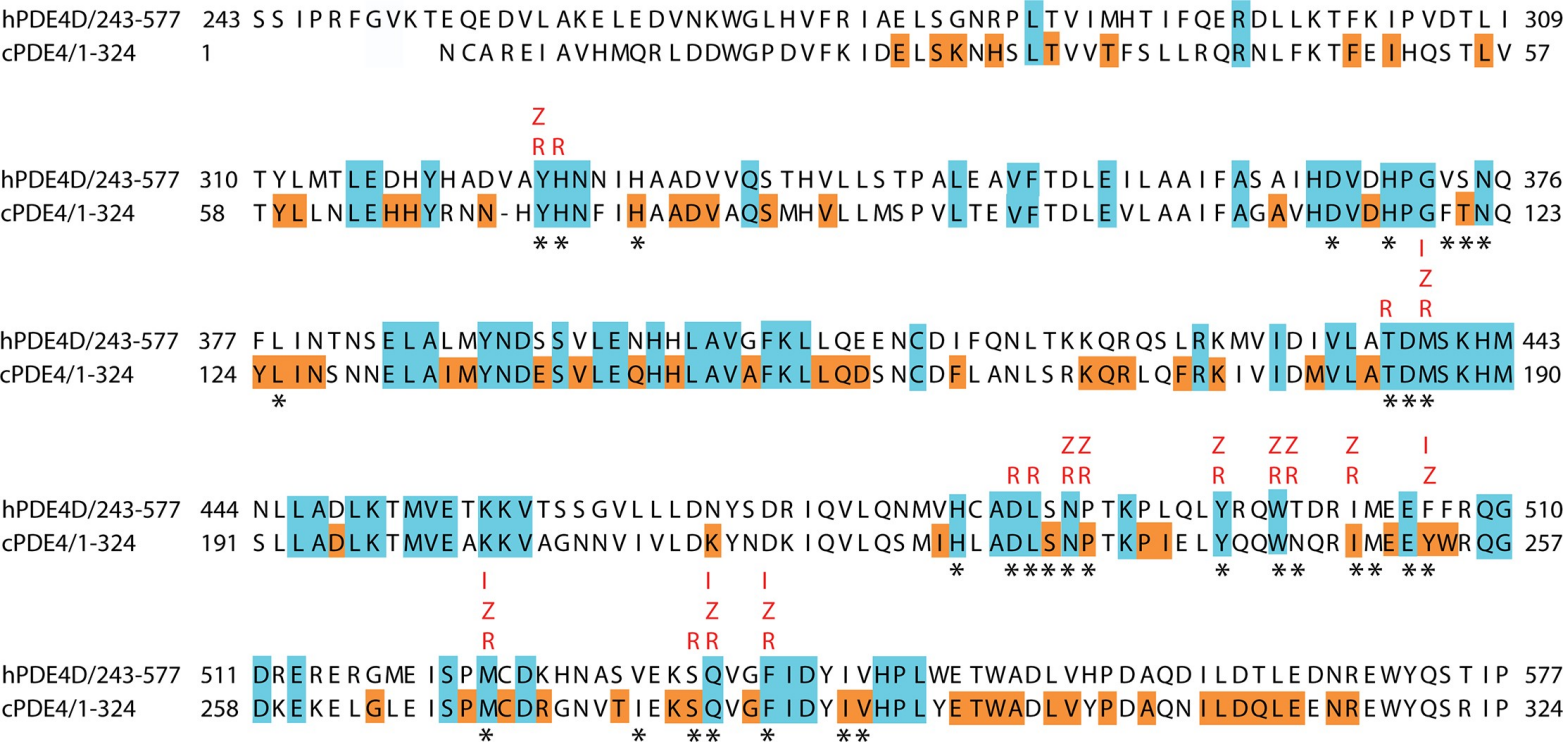
Multiple sequence alignment of human PDE4D (S243 to P577; PDB ID: 3G4L) and *C*. *elegans* PDE4 [cPDE4; numbered 1 (E289) to 324 (P608)] with amino acids that are unanimous in all PDEs we examined highlighted in blue, and additional sites that are unanimous in all nematode sequences highlighted in orange. Predicted binding sites are labelled above the residue position in red for IBMX (I), zardaverine (Z), and roflumilast (R). * denotes the 32 amino acid residues that are within 5 Å of the three ligands used in our analysis.

We next performed a multiple sequence alignment of the catalytic domain (~330 amino acids) of all PDE4 sequences in our analysis. We found that 82 (~25%) of the amino acid residues within the catalytic domain are identical in all species examined (Fig 2, colored blue). In addition, of the 32 amino acid residues that line the catalytic pocket where inhibitors bind (defined below and denoted in Fig 2 with *), 18 are unanimous sites and another 10 are 100% conserved within the nematode phylum. Also noteworthy is the observation that there are 82 residues in the catalytic domain that are identical for all nematode species we examined (Fig 2, colored orange), whereas these sites have variable amino acid residues in the non-nematode PDE4 sequences we examined. These 82 nematode-specific amino acid residues may reflect evolutionary pressure to maintain nematode-specific functional properties of PDE4 that are not shared with vertebrate PDE4 catalytic domains, and thus might result in differences in binding affinity of PDE inhibitor compounds. This observation led us to examine whether nematode PDEs differ in their ability to bind compounds known to be family-specific, high affinity inhibitors of the human PDE4 enzyme family.

### Comparative pharmacology of human and *C*. *elegans* PDE4

To evaluate the pharmacological differences of human and *C*. *elegans* PDE4, we expressed and purified the catalytic domains of *H*. *sapiens* PDE4D2 and *C*. *elegans* PDE4 (*see Materials and Methods*). We first evaluated the substrate specificity of *C*. *elegans* PDE4 to determine whether it retained the specificity for cAMP characteristic of mammalian PDE4 [55]. We found that *C*. *elegans* PDE4 has a K_m_ for cAMP of 1.7 μM (n = 2), identical to that of human PDE4 measured in our lab, and very similar to published values for human PDE4D2 [56]. In contrast, we were unable to detect significant cGMP hydrolytic activity of human or *C*. *elegans* PDE4 in order to determine the K_m_ for this substrate. We conclude that *C*. *elegans* PDE4 is a cAMP-specific PDE as is the case for human PDE4.

We next conducted dose-response experiments to compare the ability of *C*. *elegans* PDE4 and human PDE4D to be inhibited by IBMX (a non-selective PDE inhibitor), zardaverine (a PDE3/4-selective compound), or roflumilast (a potent PDE4-selective inhibitor). We observed that IBMX inhibited *C*. *elegans* PDE4 with an IC_50_ of 34.1 ± 8.7 μM (Table 2), an approximately two-fold lower value than observed for human PDE4D by us (IC_50_ value of 15.8 ± 1.7 μM) and others [57]. The Inferred Biomolecular Interactions Server (IBIS, https://www.ncbi.nlm.nih.gov/Structure/ibis/ibis.cgi; [58, 59]) predicts that IBMX binds to the following five residues in PDE4D: Met439, Phe506, Met523, Gln535, and Phe538 (Fig 2). These residues are part of the hydrophobic sub-pocket of the human PDE4D catalytic domain, and these interaction sites are also observed for zardaverine (all five sites) and roflumilast (all but Phe506; *see below*).

**Table 2:** Inhibitor dose-response relationships for human and *C*. *elegans* PDE4.

Inhibitor	Human IC_50_ (μM)	*C*. *elegans* IC_50_ (μM)	Fold difference
IBMX	15.8 ± 1.7 (n = 4)	34.1 ± 8.7 (n = 5)	2
Zardaverine	1.9 ± 0.6 (n = 5)	146 ± 34 (n = 8)	77
Roflumilast	0.0046 ± 0.0006 (n = 3)	0.73 ± 0.13 (n = 5)	159

Enzyme activity was tested over a range of inhibitor concentrations with 1 μM cAMP substrate concentration. The dose-response relationship was fit to a 3-parameter logistic equation to obtain the IC_50_ and the standard error of the mean for the indicated number of experiments.

Zardaverine is a dual PDE3/4 inhibitor, and we determined IC_50_ values for human and *C*. *elegans* of 1.9 ± 0.6 μM and 146 ± 34 μM, respectively (Table 2). The human PDE4 IC_50_ was similar to values previously reported [60]. The twelve sites predicted by IBIS to be responsible for binding of this compound to human PDE4D are: Tyr325 and Met439 (in the metal binding pocket), as well as Asn487, Pro488, Tyr495, Trp498, Thr499, Ile502, Phe506, Met523, Gln535, and Phe538 (which reside in the hydrophobic pocket; Fig 2).

Roflumilast is a potent and highly selective inhibitor of the four isozymes of human PDE4. Our analysis of roflumilast inhibition of human and *C*. *elegans* PDE4 catalytic domains reveal a 159-fold weaker affinity for the nematode enzyme, with IC_50_ values of 4.6 ± 0.56 nM and 730 ± 130 nM, respectively; Table 2). Our IC_50_ value for roflumilast binding to human PDE4D is 7-fold higher than previously reported for the recombinantly expressed human PDE4D catalytic domain [61]. IBIS predicts that roflumilast interacts with 16 residues in PDE4D (Fig 2): Tyr325, His326, Thr437, Met439, Asp484 (in the metal binding pocket), and Leu485, Asn487, Pro488, Tyr495, Trp498, Thr499, Ile502, Met523, Ser534, Gln535, and Phe538 (in the hydrophobic pocket).

### Molecular dynamics (MD) simulations to predict inhibitor binding conformations

**Fig 3:**
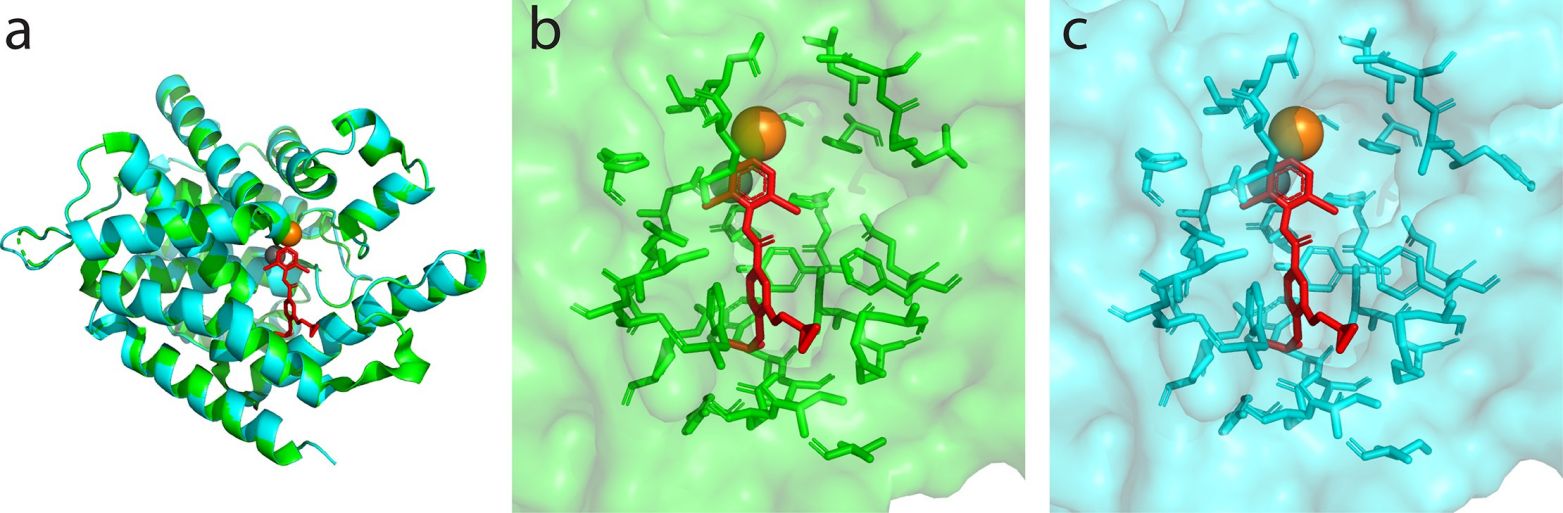
(a) Crystal structure of human PDE4D (PDB:3G4L) (green) superimposed with *C*. *elegans* PDE4 homology model (cyan), with divalent cations in orange and grey within the metal binding pocket, and roflumilast (in red) which spans the metal binding pocket and the hydrophobic pocket. (b) Human PDE4D bound to roflumilast. Surface is depicted with the 32 amino acid residues within 5 Å of the inhibitor (c) *C*. *elegans* PDE4 homology model (superimposed with roflumilast from the human PDE4D-roflumilast x-ray structure) showing the corresponding 32 nematode residues.

**Fig 4:**
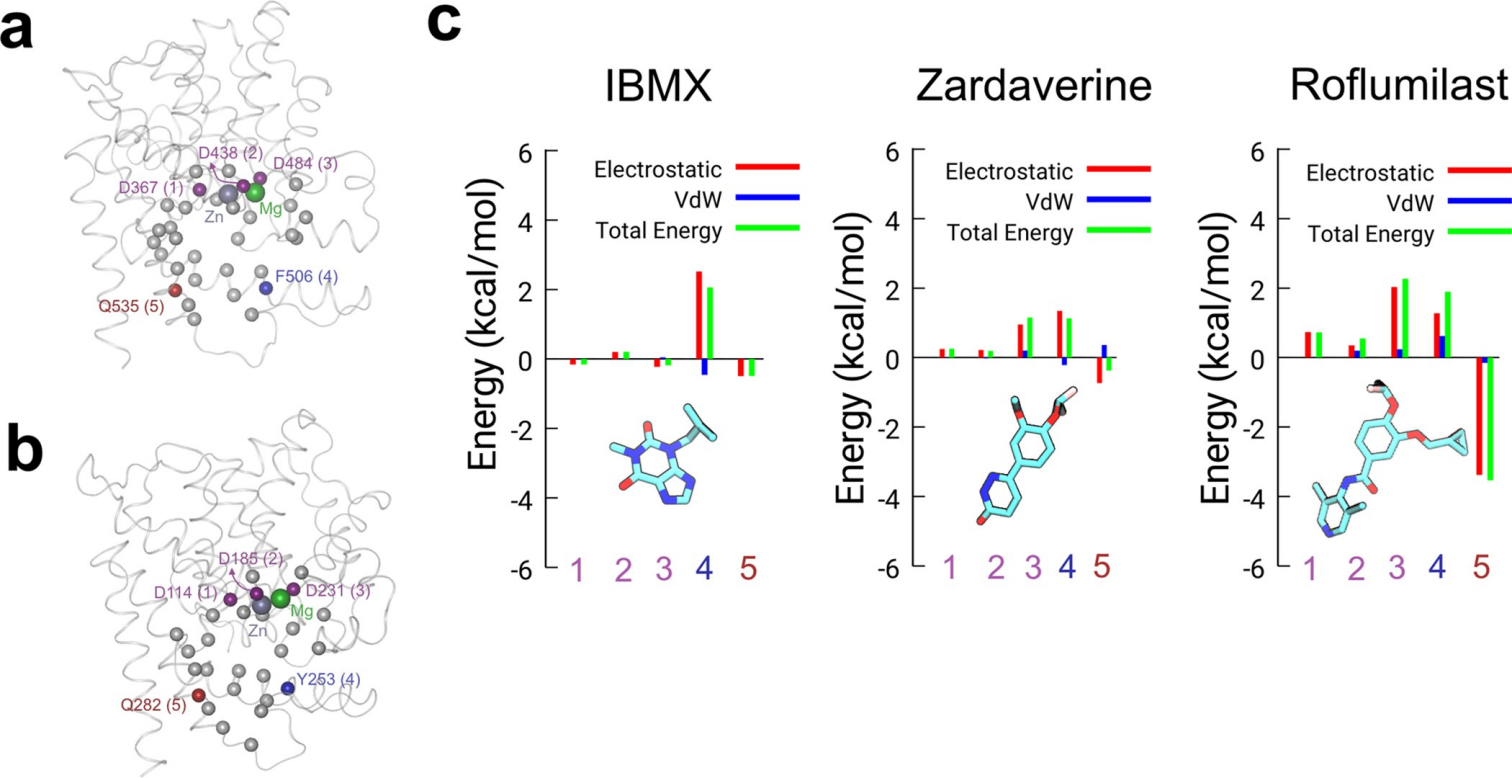
Interactions of five key C_α_-atoms of the 32 residues that interact with ligands in (a) human PDE4D and (b) *C*. *elegans* PDE4 binding pocket. The Zn and Mg ions are shown as gray and green spheres, respectively. The three Asp residues coordinating with Zn and Mg ions are highlighted by purple spheres. The Gln and Phe/Tyr residues are shown as red and blue spheres, respectively. The protein backbone is represented as ribbons, the Cα-atoms of residues of the binding pocket and ions are shown as space-filling. (c) Changes in total non-bonded interaction energy and its components for *C*. *elegans* PDE4 relative to human PDE4D are shown for selected residues in the binding pocket (labeled 1–5 in panel a and b).

To investigate the mechanistic details of differences in binding of each inhibitor, we performed two independent MD simulations (120 ns each) of human and *C*. *elegans* PDE4 with each inhibitor (IBMX, zardaverine and roflumilast), and also carried out simulations of each enzyme without inhibitors (S4 Table). The root-mean-squared-deviation (RMSD) measured relative to initial structures revealed deviations below 2 Å indicating stable structures for both enzymes (S2 Fig). Through visual analyses of these simulations, we identified 32 residues in the immediate vicinity of bound ligands (defined as within 5 Å of any of the inhibitors; Figs 3, 4A and 4B) as forming a binding pocket and then computed interaction energies of inhibitors with each of these 32 residues. In S3 and S4 Figs, we present non-bonded interaction energies (van der Waals and electrostatic) for each of the 32 residues where energies were computed based upon all atoms of each residue and of the inhibitor molecule. These analyses for human PDE4D and *C*. *elegans* PDE4 resulted from two independent sets of simulations.

From our interaction energy analyses, we identified five key residues showing differences between the human and *C*. *elegans* PDE4 (Fig 4A and 4B): (a) three conserved Asp residues (residues 367, 438, and 484 in human PDE4D corresponding to residues 114, 185, and 231 in *C*. *elegans* PDE4; purple spheres) that are critical for the coordination of the zinc and magnesium ions; (b) a conserved Gln residue (Gln535 in human PDE4D and Gln282 in *C*. *elegans* PDE4; red spheres) that stabilizes ligand binding via non-covalent interactions; and (c) a Phe residue in human PDE4D (Phe506; blue sphere) and a Tyr residue at the same position in *C*. *elegans* (Tyr253; blue sphere) that show differences in non-bonded interactions. Fig 4C presents the differences in the total non-bonded interaction energy and its components (ΔE) for *C*. *elegans* PDE4 relative to the human PDE4D at these five sites. A positive value of ΔE indicates a higher non-bonded interaction energy of a given residue with the inhibitor in *C*. *elegans* in comparison to human PDE4D, and a negative value of ΔE indicates a lower, non-bonded interaction energy. We observed positive ΔE values for the three conserved Asp residues for roflumilast and, to a lesser extent, zardaverine, indicating stronger interactions in *C*. *elegans* relative to human PDE4D. In contrast, for IBMX the ΔE values between *C*. *elegans* PDE4 and human PDE4D are comparable. For all three inhibitor complexes with *C*. *elegans* PDE4, Tyr253 showed higher nonbonded interaction energy with the inhibitors in comparison to the corresponding Phe506 residue in human PDE4D. Based on the interaction energy analysis, we observed a correlation between the change in the interaction energy at the non-conserved and conserved residue sites. Primarily for roflumilast and to a lesser extent for zardaverine and IBMX, we observed that an increase in the total non-bonded interaction energy at the non-conserved site (F506 in human vs. Y253 in *C*. *elegans*; labeled as the residue 4 in Fig 4) is correlated with a decrease in the total non-bonded interaction energy at the conserved site (Q535 in human vs. Q282 in *C*. *elegans*; labeled as the residue 5 in Fig 4). Similar correlation was observed between an increase in the total non-bonded interaction energy at the conserved site (D484 in human vs. D231 in *C*. *elegans*; labeled at the residue 3 in Fig 4) and a decrease at the conserved site (Q535 in human vs. Q282 in *C*. *elegans*; labeled as the residue 5 in Fig 4).

**Fig 5:**
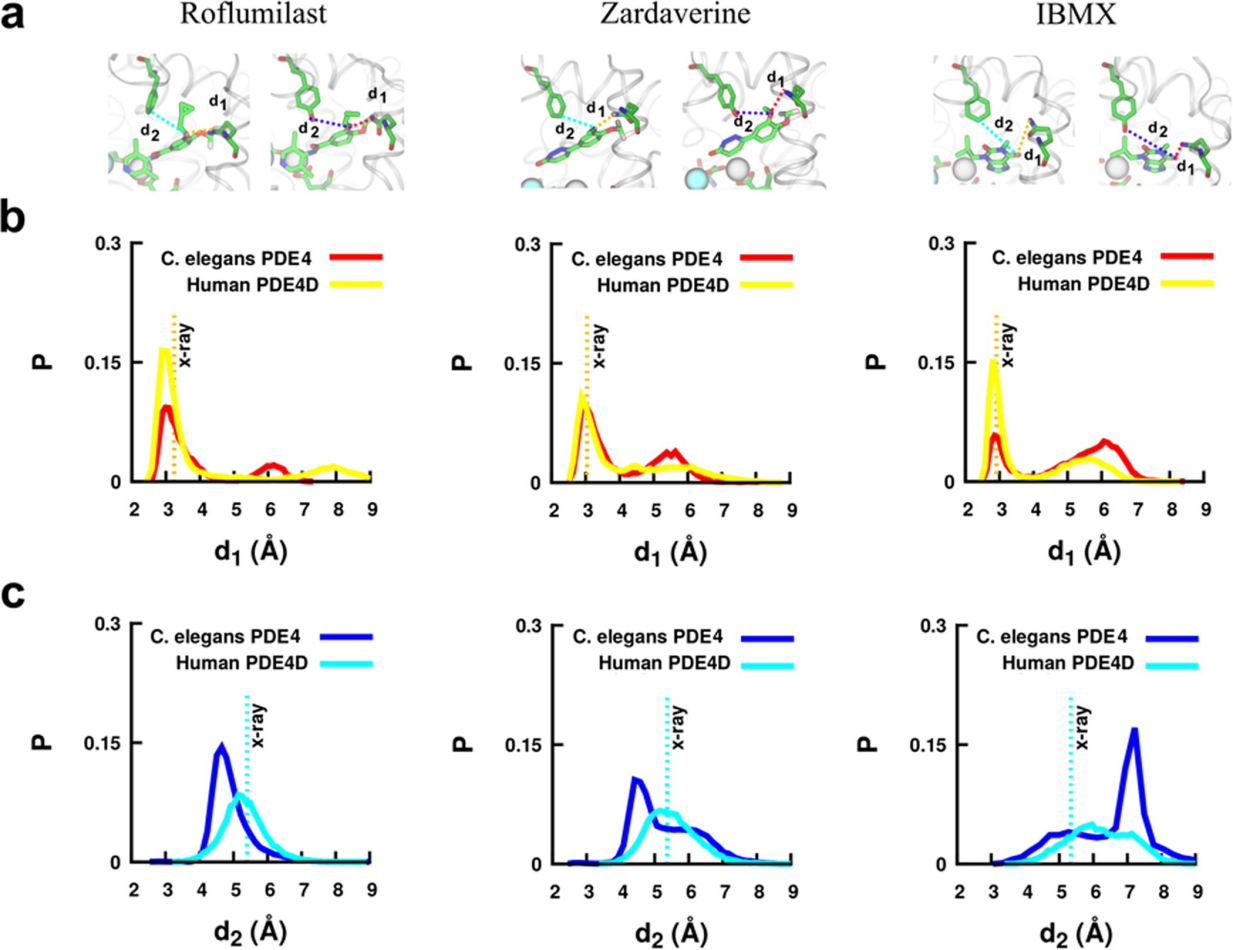
Probability (P) distributions of interatomic distances between ligand (O4 atom in roflumilast or zardaverine, or O6 atom in IBMX) and binding pocket residues. a) shows measurements of d1 [between the oxygen atom on the ligand and the N_δ_ atom on Gln535(human)/Gln282(*C*. *elegans*)] and of d2 [between the oxygen atom of the ligand and the C4 atom of Phe506 (human)/O atom on the side chain of Tyr253(*C*. *elegans*)]. b) illustrates the distributions for distance d1 for *C*. *elegans* PDE4 (red) and human PDE4D (yellow). c) shows the distributions for the distance d2 for *C*. *elegans* PDE4 (blue) and human PDE4D (cyan). Vertical dotted lines in panels b and c indicate the distances in the crystal structures of inhibitors bound to human PDE4D. The traces of these distances vs. simulation time (ns) are shown in S5–S7 Figs.

To investigate the variation in the docked positions of ligands in the binding pockets of human and *C*. *elegans* PDE4, we measured the interatomic distances between specific atoms in the ligands and the nearby Gln535(human)/Gln282(*C*. *elegans*) residues (d1 in Fig 5). For *C*. *elegans* PDE4, the distributions of d1 are bimodal (red traces in Fig 5B) for all three inhibitors, with roflumilast and zardaverine having a higher probability of being in states with d1~3Å, while for IBMX both states at d1~3Å and ~6Å are equally probable. For human PDE4D, all inhibitors show an increased probability of being in states at shorter distances (~3Å) but bimodal distributions with lower probabilities of states at larger distances are observed for zardaverine and IBMX. These observations suggest that zardaverine and IBMX are more likely to transition between two distinct states within the binding pocket in comparison to roflumilast, which appears to be stably bound, largely in a single state.

In addition, we measured the interatomic distance (d2 in Fig 5) between the oxygen atom of the inhibitors to the side-chains of Tyr253 (*C*. *elegans*) or Phe506 (human PDE4D). In *C*. *elegans* PDE4, the distance distributions are bimodal and span a larger distance range (~3–9 Å) for zardaverine and IBMX in comparison to a unimodal and narrower (~4–6 Å) distribution for roflumilast. In human PDE4D, the distributions are unimodal for roflumilast and zardaverine and binodal for IBMX, with mean values distinct from those in C. *elegans* PDE4. Overall, the measurements on these distances suggest distinct positioning of inhibitors in the proximity of Phe506 and Gln535 residues in human PDE4D and the corresponding residues Tyr253 and Gln282 in *C*. *elegans*. Specifically, roflumilast is significantly more stable than other inhibitors in the binding pocket of the human PDE4D and showed a higher non-bonded interaction energy with the Gln535 residue in human PDE4D (Fig 4C).

**Fig 6:**
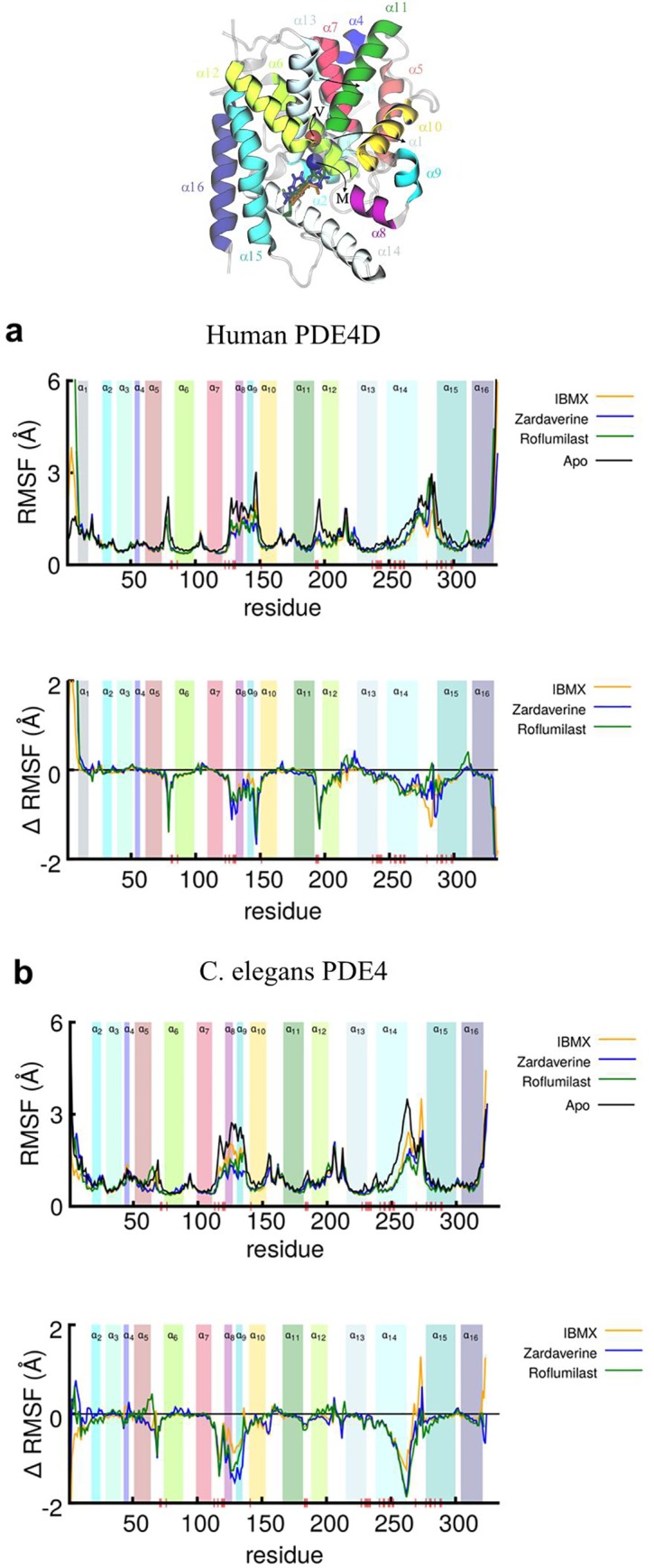
**Root-mean-squared-fluctuation (RMSF) per residue (top panel) and the change in RMSF (ΔRMSF) per residue (bottom panel) are shown for (a) human PDE4D and (b) *C*. *elegans* PDE4 complexes with IBMX, zardaverine, and roflumilast.** The superimposed structures for human PDE4D/*C*. *elegans* PDE4 along with superposition of IBMX, zardaverine, and roflumilast ligands (sticks) are shown at the top. The colored helices and vertical bars labeled α1 through α16 highlight the location of residues in the 16 helices in the catalytic domain. The Val334/81 and Met439/186 residues for human/*C*. *elegans* PDE4 are shown by red and blue spheres, respectively.

To further probe per-residue perturbations on binding of each inhibitor in both enzymes, we have computed the per-residue root-mean-squared fluctuation (RMSF) of the liganded enzyme structures (top panels in Fig 6A and 6B) and the change in per-residue RMSF relative to their unliganded apo-forms (ΔRMSF) (bottom panels in Fig 6A and 6B). Among binding pocket residues, we observed that ligand binding increased fluctuations in Val334 and Met439 in human PDE4D (corresponding to Val81 and Met186 in *C*. *elegans* PDE4). However, the residues located in loops connecting α5-α6, and α11-α12 helices are more stabilized by the ligands in human PDE4D in comparison to *C*. *elegans* PDE4. The residues located in the M loop between α8 and α9 helices are more stabilized in *C*. *elegans* PDE4 by zardaverine and roflumilast and to a lesser extent by IBMX in comparison to human PDE4D. Residue Phe506(human)/Tyr253(*C*. *elegans*) is located in α14-helix which appear more stabilized by ligands in *C*. *elegans* PDE4 in comparison to human PDE4D. Residue Gln535(human)/Gln282(*C*. *elegans*) is located in the α15-helix which is perturbed to a greater extent in human PDE4 than *C*. *elegans* PDE4 (Fig 6). The fluctuations in residues of the binding pocket as observed in the RMSF analyses are correlated with the analyses of non-bonded interaction energies.

To further investigate whether the higher flexibility of the α14-helix in human PDE4D complexes with bound ligands affects the motion of the residues belonging to the α15-helix, we calculated the dynamic cross-correlation matrix for the C_α_ atoms in all MD trajectories. For human PDE4D, the correlation matrices showed neither significant positive correlation nor significant anti-correlation between the residues of the α14-helix (highlighted by the dashed-lines in S11–S13 Figs) and the residues of the α15-helix (highlighted by the solid-lines in S11–S13 Figs). However, we find that the motion of residues in the α14 and α15 helices are marginally more correlated in *C*. *elegans* PDE4 in comparison to human PDE4D. The correlation between the α14 and α15 helices is mostly found between neighboring residues of Tyr253 (*C*. *elegans*) and Gln282 (*C*. *elegans*). We also observed (S8 Fig) significantly higher positive residue-residue (C_α_-C_α_) correlation within human PDE4D complexes with IBMX and zardaverine in comparison to *C*. *elegans* PDE4, whereas the complexes with roflumilast showed significantly lower positive correlation in human PDE4D in comparison to *C*. *elegans* PDE4. This indicates that roflumilast induces a different pattern of correlated motions in the protein backbone in comparison to IBMX and zardaverine, and comparable to those of the apo states (S9 Fig).

To better understand the effect of ligands on the protein structure outside the binding site, we identified all possible salt-bridging interactions within human PDE4D and *C*. *elegans* PDE4 (S10–S12 Figs). Qualitatively, the salt-bridging interactions are observed to occur with a lower frequency in human PDE4D in comparison to *C*. *elegans* PDE4. We also found a smaller number of salt-bridging interactions in human PDE4D complexed with roflumilast, but these salt-bridging interactions were comparatively stable for longer times during simulations. Furthermore, we identified three salt-bridge pairs conserved between human PDE4D and *C*. *elegans* PDE4 with higher occupancy number: D179-K175 (*C*. *elegans*), D204-K214 (*C*. *elegans*), and D80-K237 (*C*. *elegans*) (S10 and S11 Figs, and labeled in S13 Fig). D179-K175 (*C*. *elegans*) is an intra-helical salt-bridge in the α11-helix, and D204-K214 (*C*. *elegans*) is in the loop connecting the α12 and α13 helices. The D179-K175 (*C*. *elegans*) interaction pair in the human PDE4D complexes with ligands showed a lower occupancy in comparison to apo-human PDE4D (S12 Fig). The occupancy of the D204-K214 (*C*. *elegans*) is higher in the apo-human PDE4D in comparison to *C*. *elegans* PDE4, whereas it has a higher occupancy in *C*. *elegans* PDE4 complexes with ligands in comparison to human PDE4D complexes with ligands (S10–S13 Figs). The D80-K237 (*C*. *elegans*) salt-bridge is located near the binding pocket, the D80 residue is in the α6-helix and the K237 residue is in the loop connecting α13 and α14. We observed that the occupancy of the D80-K237 (*C*. *elegans*) salt-bridge was significantly suppressed by roflumilast in human PDE4D in comparison to zardaverine and IBMX, while the occupancy of this salt-bridge is not affected by the presence of IBMX and zardaverine (S10–S13 Figs). Both dynamic cross-correlation analysis and salt-bridging interactions revealed allosteric effects of each ligand on the protein structure. Unlike IBMX and zardaverine, roflumilast induced distinct patterns of structural perturbations outside of the binding pocket for human PDE4D compared with *C*. *elegans* PDE4. Specifically, we observed lower residue-residue correlations for roflumilast in comparison to zardaverine and IBMX in human PDE4D in comparison to *C*. *elegans* PDE4. While we observed overall a smaller number of salt-bridging interactions in human PDE4D in comparison to *C*. *elegans* PDE4, the salt-bridging interactions in human PDE4D were significantly more stable for roflumilast. In contrast, roflumilast significantly perturbed some salt-bridging interactions (D88-K237) more than zardaverine and IBMX in *C*. *elegans* PDE4. Therefore, we suggest that modulation of salt-bridging interactions could be one of the factors that contribute to an altered binding affinity of an inhibitor among different protein isoforms. Taken together, these structural analyses provide a molecular basis for better understanding differential binding of inhibitory compounds in the human PDE4 versus *C*. *elegans* PDE4 catalytic domain.

## Discussion

### Nematode PDEs are evolutionarily divergent

In order to evaluate potential differences in pharmacological properties between vertebrate and nematode PDEs, we first established which PDE genes are present in nematodes. Our results indicate that the genomes of the nematode phylum, regardless of clade, encode six orthologs of vertebrate PDEs: PDE1, PDE2, PDE3, PDE4, PDE8, and PDE10. Unlike the vertebrate orthologs of the PDE1, PDE3, PDE4, and PDE8 families which consist of multiple isozymes [25], all of the nematodes species we studied encode only a single gene for each enzyme family. This is consistent with one or more genome duplication events that occurred after the last common ancestor of nematodes and vertebrates [62, 63].

We originally expected that nematode PDEs would be more closely related to the PDEs found in other protostomes, such as *C*. *telata*, *I*. *scapularis*, *D*. *pulex*, and *D*. *melanogaster*. However, in the case of nematode PDE2, PDE3, PDE4, and PDE8, the results, shown in S1 Fig, do not support this hypothesis. These nematode PDE families are separated cladistically from all other species in our analysis, suggesting a higher degree of divergence and thus greater differences in their protein sequence compared to other organisms. Furthermore, the observation of 82 conserved residues in all nematode PDE4 catalytic domain sequences at sites that are variable in non-nematode PDE4 orthologs may be indicative of nematode-specific structural differences in the catalytic domain that underlie the observed differences in binding affinity of PDE4-selective inhibitor compounds for human and *C*. *elegans* PDE4 (Table 2).

### PDE4-selective inhibitors reveal differences in their affinity for nematode and human PDE4

Based on previous research suggesting that PDE4 in *C*. *elegans* is expressed in the nervous system and regulates cAMP levels in intrasynaptic pools that regulate locomotion [18], we chose to characterize nematode PDE4 by comparing its pharmacological sensitivity to a non-selective inhibitor (IBMX) as well as two compounds identified as being selective inhibitors of human PDE4.

In the case of IBMX, the modest decrease in binding affinity to *C*. *elegans* PDE4 compared to human PDE4D (Table 2) indicates that there is little difference in how human PDE4D and *C*. *elegans* PDE4 bind this inhibitor. Supporting this conclusion, IBIS analysis predicts that IBMX interacts with only five residues within the catalytic domain, all of which are contained within the hydrophobic sub-pocket of PDE4. Four of these five interaction sites are conserved not only between humans and nematodes but across all the species we analyzed (Fig 2). The one difference we identified was the Phe506 in human PDE4D that is substituted with Tyr253 in *C*. *elegans*; notably, this tyrosine residue is conserved in all nematode species examined in this study (Fig 2).

In contrast, zardaverine has a 77-fold decreased affinity for *C*. *elegans* PDE4 compared with human PDE4D (Table 2). IBIS analysis predicted that zardaverine interacts with 12 residues in both the ion binding and hydrophobic sub-pockets of PDE4, of which ten are conserved between human and nematode PDE4. Two significant differences in the zardaverine binding site of human and *C*. *elegans* PDE4 were identified at residues Thr499 (Asn246 in *C*. *elegans*) and Phe506 (Tyr253 in *C*. *elegans*) which may contribute to the lower binding affinity of zardaverine for the nematode enzyme. Notably, *C*. *elegans* PDE4 binds zardaverine with lower affinity than IBMX (Table 2); this may reflect destabilizing interactions of zardaverine with residues comprising the ion binding pocket, whereas IBMX interactions are confined to the hydrophobic pocket (see also next section).

Roflumilast, a potent and selective inhibitor of human PDE4, showed a 159-fold decrease in affinity for *C*. *elegans* PDE4 compared with its high affinity for human PDE4 (Table 2). The sixteen residues predicted by IBIS to interact with roflumilast span both the ion binding and hydrophobic sub-pocket of the catalytic domain of PDE4 (Fig 3). Interestingly, while the IBIS analysis supports a role for Phe506 (Tyr253 in *C*. *elegans*) in stabilizing IBMX and zardaverine, it does not predict a role in the binding of roflumilast. However, our MD results (see below) suggest that this position is indeed an important factor in the affinity of PDE4 for roflumilast.

### Atomistic simulations provide insight into altered pharmacological properties

As described above, the evolutionary analysis supported substantial differences in the primary sequence of nematode and vertebrate PDEs and pharmacological results revealed significant changes in binding affinities of compounds that were designed for human PDE4D. To gain further insights into differences in the ligand binding sites of *C*. *elegans* PDE4 and human PDE4D that could explain the reduced affinity of *C*. *elegans* PDE4 for compounds optimized as human PDE inhibitors, we used homology models (Fig 3) and all-atom explicit-solvent MD simulations. The use of homology models has been successfully used in previous studies to identify amino acid residues that are responsible for differences in binding of inhibitors to PDE5 and PDE6 [64]. From the 32 amino acid residues that we defined as constituting the inhibitor-binding site, only five sites differed between the two enzymes and four of those were conservative substitutions that preserved the polar or hydrophobic nature at its position and thus are unlikely to drastically change the binding conformation or energy. However, we observed differences in nonbonded interaction energies due to the movement of inhibitors in the vicinity of residue Phe506 in *H*. *sapiens* (corresponding to Tyr253 in *C*. *elegans*). Overall, this Tyr residue contributes significantly more total non-bonded interaction energy than the Phe in the same position for all three inhibitors (S3 and S4 Figs), likely due to the hydrogen bonding that results from the addition of a hydroxyl group at the 4-C of the aromatic ring.

For IBMX, the Phe to Tyr substitution appears to have little impact on the overall binding of IBMX to either human PDE4D or *C*. *elegans* PDE4. This is likely a result of IBMX interacting solely with the hydrophobic pocket of PDE4, as suggested by IBIS. Despite the polar Tyr residue coordinating to the ketone at position 6 in the purine ring of IBMX in *C*. *elegans*, our MD results indicate that the interactions and conformation of IBMX are very similar in the two PDE4 catalytic domains. This is consistent with the observed similarity in IC_50_ values for IBMX with the two enzymes.

In contrast, the MD simulations suggest that the binding conformation of zardaverine or roflumilast are altered as a result of the substitution of Tyr253 for Phe506 at this site in the binding pocket of the two enzymes, consequently inducing a different pattern of correlated motions in the protein backbone. In *C*. *elegans*, the hydroxyl group of the Tyr residue coordinates strongly with the methoxyphenyl and cyclopropylmethoxyl group of zardaverine and roflumilast, respectively. This increase in energy contribution appears to result in a displacement of both ligands away from the hydrophobic sub-pocket that further disrupts stabilization by the conserved glutamine residues (Gln535 in human PDE4; Fig 4C). It has been previously reported that Tyr495, Phe506, Gln535 are critical for stabilizing PDE4 inhibitors [31]. This disruption of the desired binding conformation in *C*. *elegans* could partially explain the reduced IC_50_ values for these two compounds. While per-residue fluctuations are also found to be correlated with non-bonded interaction energy analyses, other analyses (e.g. interatomic distances and residue-residue correlations) suggest that, among the three inhibitors, roflumilast is more stable in the binding pockets and induces a distinct pattern of correlated motions in comparison to zardaverine and IBMX. In summary, these MD analyses highlight the importance of considering not only differences in residue substitutions (e.g. Tyr253 in *C*. *elegans* vs. Phe506 in *H*. *sapiens*) but also allosteric perturbations and overall inhibitor stabilization of catalytic domain conformation in future efforts to design and optimize nematode-specific PDE inhibitor compounds using *in silico* approaches such as virtual screening and fragment-based drug design [65, 66].

## Conclusions

In conclusion, we have combined information on the evolution of the PDE superfamily, targeted pharmacological comparisons of inhibitor binding profiles, and MD simulations to support the hypothesis that the nematode PDE enzyme family differs sufficiently from the vertebrate PDE orthologs to validate the feasibility of developing PDE inhibitor compounds as potent and selective anthelmintics/nematicides. While analysis of the differences in the amino acid sequence or structure-activity relationships for selected PDE inhibitor compounds did not immediately identify which sites of interaction may have been disrupted in *C*. *elegans* PDE4 for inhibitors designed for human PDE4D, MD simulations revealed the importance of Phe506 (human)/Tyr253 (*C*. *elegans*) substitution and demonstrated that changes in the conformation of the catalytic domain may collectively lead to inhibitor discrimination in the binding pocket, based on the following analyses: (1) non-bonded interaction energy analysis; (2) changes in the ligand orientation in the binding pocket; (3) RMSF analysis; (4) cross correlation analysis; and (5) salt-bridge interaction analysis. Collectively, our results indicate that future efforts to discover inhibitor compounds specifically targeting nematode PDE4 must take into consideration not only the molecular architecture of the inhibitor binding site, but also the conformational dynamics of the entire catalytic domain of the enzyme. Insights gained from this study will advance efforts to rationally design inhibitor compounds that selectively and potently inhibit plant and animal parasitic nematode PDEs to disrupt their lifecycle, thereby enhancing public health and agricultural productivity.

## Supporting information

S1 Table Protein model databases used in this study. (PDF)

S2 Table BLAST results from the phylogenomic pipeline after elimination of redundant sequences. The left column contains the labels used for the phylogenetic tree in S1 Fig, and the right column contains either the protein accession number or the identifying header from the protein databases included in the analysis. Sequences in **bold** were removed based on criteria described in *Materials and Methods*.
(PDF)

S3 Table Table of correspondence for the amino acid residue numbers of human PDE4D (PDB IDs 3G4L, 1MKD, and 1ZKN) and for the corresponding *C*. *elegans* PDE4 residues. The table lists the amino acids present in the human and *C*. *elegans* PDE4 catalytic domain at the given position, as well as the residue number for each of the four different protein sequences used. *C*. *elegans* residue begins at Asn285 and ends at Pro608 (Uniprot ID: S6FCW6). Blank cells denote no amino acid residue present at that position.
(PDF)

S4 Table Details of MD simulations. (PDF)

S1 Fig Phylogeny of the catalytic domain of putative PDE genes. Bootstrap analysis was run 100 times and any support values less than 50 were removed from the tree for clarity.
(PDF)

S2 Fig The traces of root-mean-squared-deviation (RMSD) vs. simulation time (ns) for PDE4D and *C. elegans* PDE4. (a and b) Two independent simulation runs for complexes of human PDE4D and *C. elegans* PDE4 with IBMX, zardaverine, or roflumilast. (c) RMSD traces of three independent simulation runs of apo-PDE4D and apo-*C*. *elegans* PDE4.
(PDF)

S3 Fig The nonbonded interaction energy analysis between residues in the inhibitor binding pocket of PDE4D and *C*. *elegans* PDE4 for the first simulation run. See Fig 3 and Fig 4A and 4B for depictions of the binding pocket and the 32 residues analyzed with bound (a) IBMX, (b) zardaverine, and (c) roflumilast. Amino acid residues in blue text denote residues that differ between human and *C*. *elegans* PDE4 sequences.
(PDF)

S4 Fig The nonbonded interaction energy analysis between residues in the inhibitor binding pocket of PDE4D and *C*. *elegans* PDE4 for the second simulation run. (a) IBMX, (b) zardaverine, and (c) roflumilast. Amino acid residues in blue text denote residues that differ between human and *C*. *elegans* PDE4.
(PDF)

S5 Fig Interatomic distances between C4 atom of F506(human)/O atom on the side chain of Y253(*C. elegans*) (blue dashed line, labeled 1) or the Nδ atom of Q369/Q282 (red dashed line, labeled 2) and the O6 oxygen of IBMX bound to human PDE4D or *C. elegans* PDE4 obtained from two independent MD simulation runs, (a) run 1 and (b) run 2.
(PDF)

S6 Fig Interatomic distances between C4 atom of F506(human)/O atom on the side chain of Y253(*C*. *elegans*) (blue dashed line, labeled 1) or the N_δ_ atom of Q369/Q282 (red dashed line, labeled 2) and the O4 oxygen of zardaverine bound to human PDE4D or *C*. *elegans* PDE4 obtained from two independent MD simulation runs, (a) run 1 and (b) run 2.
(PDF)

S7 Fig Interatomic distances between C4 atom of F506(human)/O atom on the side chain of Y253(*C. elegans*) (blue dashed line, labeled 1) or the Nδ atom of Q369/Q282 (red dashed line, labeled 2) and the O4 oxygen of roflumilast bound to human PDE4D or *C. elegans* PDE4 obtained from two independent MD simulation runs, (a) run 1 and (b) run 2.
(PDF)

S8 Fig Dynamic cross correlation matrices calculated for the C_α_ atoms of human PDE4D and *C*. *elegans* PDE4 complexed with IBMX (a), zardaverine (b), and roflumilast (c). Residues in the α14 and α15 helices are shown by areas between dashed-lines and solid-lines, respectively. Red tick-marks on the axes represent the 32 residues in the binding site (as depicted in Fig 4A and 4B). The color scheme ranges from anticorrelation (-1.0, blue), no correlation (0, green), and positive correlation (+1.0, red). Values are the average for the two independent simulation runs.
(PDF)

S9 Fig Dynamic cross correlation matrices calculated for the C_α_ atoms of human PDE4D and *C*. *elegans* PDE4 in their apo state. Color scheme is the same as for S8 Fig. Panels a-c represent three independent simulations.
(PDF)

S10 Fig Key salt-bridging interactions are shown based upon the first set of MD simulations of human PDE4D and *C. elegans* PDE4 with IBMX (a), zardaverine (b), and roflumilast (c). Three conserved salt-bridges are labeled in blue.
(PDF)

S11 Fig Data similar to S10 Fig are shown for a second set of MD simulations with the three inhibitors. (PDF)

S12 Fig Data similar to S10 Fig are shown for three independent sets of MD simulations of apo human PDE4D and apo *C*. *elegans* PDE4. (PDF)

S13 Fig *C*. *elegans* PDE4 catalytic domain illustrating three conserved salt-bridges. Residues participating in each salt-bridge are colored and labeled. The three inhibitors are shown as sticks.
(PDF)
