# Supplementary material for: Pharmacological and molecular dynamics analyses of differences in inhibitor binding to human and nematode PDE4: Implications for management of parasitic nematodes
Source: PLoS One. 2019 Mar 27;14(3):e0214554. doi: 10.1371/journal.pone.0214554 (PMC6436744; doi:10.1371/journal.pone.0214554)
Supplement: S1 Table — (PDF) [file pone.0214554.s001.pdf]

S1 Table. Protein model databases used in this study.

Organism	Classification	Protein Database
Branchiostoma belcheri (Belcher's Lancet)	Chordata: Cephalochordata	NCBI *Branchiostoma belcheri* Annotation Release 100
Brugia malayi (animal parasitic nematode)	Nematoda: Clade III	Wormbase PRJNA10729.WS261
Bursaphelenchus xylophilus (plant parasitic nematode)	Nematoda: Clade IV	Wormbase PRJEA64437.WBPS9
C. elegans (free-living nematode)	Nematoda: Clade V	Wormbase PRJNA13758.WS261
Capitella telata (polycheate worm)	Annilida: Polycheata	JGI v1.0
Ciona Savignyi (sea squirt)	Chordata: Tunicata	Ensembl Database, CSAV2.0
Danio rerio (zebrafish)	Chordata: Vertebrata	NCBI *Danio rerio* Annotation Release 106
Daphnia pulex (water flea)	Arthropoda: crustacea	JGI v1.1
Drosophila melanogaster (fruit fly)	Arthropoda: Hexapoda	Ensembl Database, BDGP6
Globodera pallida (plant parasitic nematode)	Nematoda: Clade IV	Wormbase PRJEB123.WBPS9
Globodera rostochiensis (plant parasitic nematode)	Nematoda: Clade IV	Wormbase PRJEB13504.WBPS9
Homo sapien (human)	Chordata: Vertebrata	NCBI *Homo sapiens* Annotation Release 108
Ixodes scapularis (black-legged tick)	Arthropoda: Arachnida	Vector Base IscaW1.6
Meloidogyne floridensis (plant parasitic nematode)	Nematoda: Clade IV	Wormbase PRJEB6016.WBPS9
Meloidogyne hapla (plant parasitic nematode)	Nematoda: Clade IV	Wormbase PRJNA29083.WBPS9
Meloidogyne incognita (plant parasitic nematode)	Nematoda: Clade IV	Wormbase PRJEA28837.WBPS9
Onchocerca volvulus (human parasitic nematode)	Nematoda: Clade III	Wormbase PRJEB513.WS261
P. pacificus (free-living nematode)	Nematoda: Clade V	Wormbase PRJNA12644.WS261
Saccoglossuc kowalevskii (acorn worm)	Hemichordata	NCBI *Saccoglossus kowalevskii* Annotation Release 101
Strongyloides rattii (animal parasitic nematode)	Nematoda: Clade IV	Wormbase PRJEB125.WS261
