# Supplementary material for: Pharmacological and molecular dynamics analyses of differences in inhibitor binding to human and nematode PDE4: Implications for management of parasitic nematodes
Source: PLoS One. 2019 Mar 27;14(3):e0214554. doi: 10.1371/journal.pone.0214554 (PMC6436744; doi:10.1371/journal.pone.0214554)
Supplement: S2 Table — The left column contains the labels used for the phylogenetic tree in S1 Fig, and the right column contains either the protein accession number or the identifying header from the protein databases included in the analysis. Sequences in bold were removed based on criteria described in Materials and Methods. (PDF) [file pone.0214554.s002.pdf]

S2 Table. BLAST results from the phylogenomic pipeline after elimination of redundant sequences. The left column contains the labels used for the phylogenetic tree in Figure S1, and the right column contains either the protein accession number or the identifying header from the protein databases included in the analysis. Sequences in bold were removed based on criteria described in Materials and Methods.

Pipeline Designation	Accession #/Database ID
Branchiostoma|10132	XP_019621737.1
Branchiostoma|10134	XP_019621741.1
Branchiostoma|16467	XP_019628650.1
Branchiostoma|18907	XP_019631282.1
Branchiostoma|18910	XP_019631273.1
Branchiostoma|18913	XP_019631280.1
Branchiostoma|18915	XP_019631279.1
Branchiostoma|18916	XP_019631277.1
Branchiostoma|18917	XP_019631281.1
Branchiostoma|18918	XP_019631278.1
Branchiostoma|18919	XP_019631284.1
Branchiostoma|18921	XP_019631289.1
Branchiostoma|18922	XP_019631287.1
Branchiostoma|18924	XP_019631291.1
Branchiostoma|18927	XP_019631292.1
Branchiostoma|20407	XP_019633000.1
Branchiostoma|20409	XP_019633002.1
Branchiostoma|23511	XP_019636328.1
Branchiostoma|25581	XP_019639056.1
Branchiostoma|25582	XP_019639057.1
Branchiostoma|27175	XP_019640468.1
Branchiostoma|29321	XP_019643058.1
Branchiostoma|30608	XP_019644260.1
Branchiostoma|30611	XP_019644259.1
Branchiostoma|32476	XP_019646430.1
**Branchiostoma|5613**	**XP_019616527.1**
Branchiostoma|6430	XP_019617478.1
Branchiostoma|7052	XP_019618078.1
Branchiostoma|7053	XP_019618073.1
Branchiostoma|7055	XP_019618075.1
Branchiostoma|7780	XP_019619259.1
Branchiostoma|7781	XP_019619261.1
Brugia|10983	>Bm7204 wormpep=BM38553 gene=WBGene00227465 locus=Bma-pde-6 status=Confirmed uniprot=A0A0H5S4Q5
Brugia|5205	>Bm2519 wormpep=BM20904 gene=WBGene00222780 locus=Bma-pde-5 status=Partially_confirmed uniprot=A0A0I9N6W0
Brugia|5902	>Bm3084 wormpep=BM44778 gene=WBGene00223345 locus=Bma-pde-3 status=Partially_confirmed uniprot=A0A0H5S0K1
Brugia|8657	>Bm5306 wormpep=BM41506 gene=WBGene00225567 status=Partially_confirmed uniprot=A0A0J9Y1Y6
Brugia|8819	>Bm5437 wormpep=BM43536 gene=WBGene00225698 locus=Bma-pde-4 status=Partially_confirmed uniprot=A0A0H5S496
Brugia|8948	>Bm5547 wormpep=BM40429 gene=WBGene00225808 locus=Bma-pde-1 status=Confirmed uniprot=A0A0I9N7C3
Bursaphelenchus|10439	>BXY_0888800.1 transcript=BXY_0888800.1 gene=BXY_0888800
Bursaphelenchus|12230	>BXY_0264400.1 transcript=BXY_0264400.1 gene=BXY_0264400
Bursaphelenchus|15631	>BXY_1697300.1 transcript=BXY_1697300.1 gene=BXY_1697300
Bursaphelenchus|2916	>BXY_1002500.1 transcript=BXY_1002500.1 gene=BXY_1002500
Bursaphelenchus|5762	>BXY_1645400.1 transcript=BXY_1645400.1 gene=BXY_1645400

Pipeline Designation	Accession #/Database ID
Drosophila|9323	[Source:FlyBase;Acc:FBgn0259171]
GloboderaPallida/rostonchiensis|1406	>GROS_g01406.t1 transcript=GROS_g01406.t1 gene=GROS_g01406
GloboderaPallida/rostonchiensis|510	>GROS_g00510.t1 transcript=GROS_g00510.t1 gene=GROS_g00510
GloboderaPallida/rostonchiensis|5161	>GROS_g05161.t1 transcript=GROS_g05161.t1 gene=GROS_g05161
GloboderaPallida/rostonchiensis|6470	>GROS_g06470.t1 transcript=GROS_g06470.t1 gene=GROS_g06470
GloboderaPallida/rostonchiensis|7797	>GROS_g07797.t1 transcript=GROS_g07797.t1 gene=GROS_g07797
**GloboderaPallida/rostonchiensis|8612**	**>GROS_g08612.t1 transcript=GROS_g08612.t1 gene=GROS_g08612**
**GloboderaPallida/rostonchiensis|8613**	**>GROS_g08613.t1 transcript=GROS_g08613.t1 gene=GROS_g08613**
GloboderaPallida|11976	>GPLIN_001018100 transcript=GPLIN_001018100 gene=GPLIN_001018100
**GloboderaPallida|14144**	**>GPLIN_001591800 transcript=GPLIN_001591800 gene=GPLIN_001591800**
**GloboderaPallida|3887**	**>GPLIN_001325100 transcript=GPLIN_001325100 gene=GPLIN_001325100**
GloboderaPallida|676	>GPLIN_000139100 transcript=GPLIN_000139100 gene=GPLIN_000139100
GloboderaPallida|7042	>GPLIN_000164100 transcript=GPLIN_000164100 gene=GPLIN_000164100
GloboderaPallida|9957	>GPLIN_001023500 transcript=GPLIN_001023500 gene=GPLIN_001023500
Homo|101680	NP_001001570.1
Homo|101681	NP_002597.1
Homo|101682	NP_001001567.1
Homo|101683	NP_001001583.1
Homo|101684	NP_001001574.1
Homo|101685	NP_001001578.1
Homo|101686	NP_001001569.1
**Homo|101687**	**XP_011527902.1**
**Homo|101688**	**XP_016883856.1**
Homo|101691	NP_001001577.1
Homo|101695	XP_011527900.1
Homo|101696	NP_001302462.1
Homo|16513	NP_001070665.1
Homo|16514	NP_058649.3
Homo|16648	NP_001245241.1
Homo|16654	XP_016859791.1
Homo|16658	XP_016859789.1
Homo|16659	NP_001245242.1
Homo|16660	NP_001245243.1
Homo|16661	XP_011509625.1
Homo|16662	XP_016859783.1
Homo|16663	XP_011509627.1
Homo|16664	XP_011509628.1
Homo|16665	XP_011509626.1
Homo|25934	XP_011511775.1
Homo|25935	XP_011511776.1
Homo|25945	XP_011511779.1
Homo|25946	XP_016863777.1
Homo|28912	NP_001074.2

Bursaphelenchus|6750	>BXY_0576000.1 transcript=BXY_0576000.1 gene=BXY_0576000
Caenorhabditis|16934	>R08D7.6a wormpep=CE44867 gene=WBGene00011146 locus=pde-2 status=Confirmed uniprot=P30645
Caenorhabditis|17507	>R153.1a wormpep=CE02038 gene=WBGene00020114 locus=pde-4 status=Partially_confirmed uniprot=Q22000
Caenorhabditis|17509	>R153.1c wormpep=CE32060 gene=WBGene00020114 locus=pde-4 status=Confirmed uniprot=Q22000
Caenorhabditis|17510	>R153.1d wormpep=CE33438 gene=WBGene00020114 locus=pde-4 status=Confirmed uniprot=Q22000
Caenorhabditis|17513	>R153.1g wormpep=CE39396 gene=WBGene00020114 locus=pde-4 status=Confirmed uniprot=Q2V4U0
Caenorhabditis|18059	>T04D3.3a wormpep=CE16340 gene=WBGene00011433 locus=pde-1 status=Partially_confirmed uniprot=O18696
Caenorhabditis|18060	>T04D3.3b wormpep=CE42925 gene=WBGene00011433 locus=pde-1 status=Partially_confirmed uniprot=B5BM35
Caenorhabditis|26550	>Y95B8A.10a wormpep=CE23151 gene=WBGene00022389 locus=pde-6 status=Partially_confirmed uniprot=Q9N2V9
Caenorhabditis|4022	>C32E12.2 wormpep=CE45692 gene=WBGene00016328 locus=pde-5 status=Partially_confirmed uniprot=P91119
Caenorhabditis|6542	>E01F3.1a wormpep=CE32847 gene=WBGene00008443 locus=pde-3 status=Partially_confirmed uniprot=Q8I0P7
Caenorhabditis|6544	>E01F3.1c wormpep=CE43353 gene=WBGene00008443 locus=pde-3 status=Partially_confirmed uniprot=Q8I0P7
Caenorhabditis|6545	>E01F3.1d wormpep=CE43321 gene=WBGene00008443 locus=pde-3 status=Partially_confirmed uniprot=Q8I0P7
Caenorhabditis|6550	>E01F3.1i wormpep=CE48629 gene=WBGene00008443 locus=pde-3 status=Confirmed uniprot=U4PEM9
Capitella|1365	>jgi|Capca1|180250|estExt_Genewise1Plus.C_10235
Capitella|18691	>jgi|Capca1|201790|fgenesh1_pg.C_scaffold_380000021
Capitella|19499	>jgi|Capca1|136586|e_gw1.223.32.1
Capitella|2085	>jgi|Capca1|125768|e_gw1.485.8.1
**Capitella|2408**	**>jgi|Capca1|188739|fgenesh1_pg.C_scaffold_86000048**
Capitella|2412	>jgi|Capca1|219957|estExt_fgenesh1_pg.C_860050
Capitella|3235	>jgi|Capca1|160338|estExt_Genewise1.C_6150014
Capitella|3891	>jgi|Capca1|155235|estExt_Genewise1.C_2640047
Capitella|6390	>jgi|Capca1|169631|estExt_Genewise1Plus.C_2620007
Capitella|6874	>jgi|Capca1|103680|e_gw1.20.32.1
Capitella|9455	>jgi|Capca1|124844|e_gw1.45.93.1
Capitella|9939	>jgi|Capca1|109907|e_gw1.146.40.1
Ciona|11917	>ENSCSAVP00000011923.1 pep reftig:CSAV2.0:reftig_58:431008:443925:1 gene:ENSCSAVG00000007004.1 transcript:ENSCSAVT00000012061.1
Ciona|14913	>ENSCSAVP00000014919.1 pep reftig:CSAV2.0:reftig_58:1319061:1324140:1 gene:ENSCSAVG00000008735.1 transcript:ENSCSAVT00000015093.1
**Ciona|15016**	**>ENSCSAVP00000015022.1 pep reftig:CSAV2.0:reftig_26:1398544:1401824:1 gene:ENSCSAVG00000008803.1 transcript:ENSCSAVT00000015196.1**
Ciona|17585	>ENSCSAVP00000017592.1 pep reftig:CSAV2.0:reftig_30:3374112:3385873:-1 gene:ENSCSAVG00000010358.1 transcript:ENSCSAVT00000017784.1
Ciona|17587	>ENSCSAVP00000017594.1 pep reftig:CSAV2.0:reftig_30:3374598:3385870:-1 gene:ENSCSAVG00000010358.1 transcript:ENSCSAVT00000017786.1
**Ciona|17620**	**>ENSCSAVP00000017627.1 pep reftig:CSAV2.0:reftig_48:1416577:1419477:-1 gene:ENSCSAVG00000010375.1 transcript:ENSCSAVT00000017819.1**

Homo|31197	XP_016865054.1
Homo|31200	NP_001098101.1
Homo|31202	NP_001184147.1
Homo|31206	XP_016865058.1
Homo|31207	NP_001184148.1
Homo|31208	NP_001184149.1
Homo|31211	NP_001184151.1
Homo|31213	NP_001184152.1
**Homo|31687**	**XP_016865496.1**
Homo|31688	XP_006714788.1
Homo|31689	XP_011542002.1
Homo|31691	NP_003710.1
Homo|31692	NP_001025023.1
Homo|31693	NP_001025025.1
Homo|31694	NP_001025022.1
Homo|31697	NP_001025024.1
Homo|33725	XP_016865061.1
Homo|33726	NP_000431.2
Homo|39234	XP_011534057.1
Homo|39236	XP_005266988.1
Homo|40028	XP_011533689.2
Homo|40029	NP_001124162.1
Homo|40036	XP_016865683.1
**Homo|40037**	**XP_016865686.1**
Homo|41161	NP_001177987.2
Homo|41162	XP_016867753.1
Homo|41167	NP_001308984.1
Homo|41168	NP_001308988.1
Homo|4281	NP_001284370.1
Homo|4283	NP_002591.2
Homo|4285	NP_001032417.1
Homo|4286	XP_016856934.1

**Ciona|17621**	**>ENSCSAVP00000017628.1 pep reftig:CSAV2.0:reftig_48:1425365:1426174:-1 gene:ENSCSAVG00000010376.1 transcript:ENSCSAVT00000017820.1**
**Ciona|17622**	**>ENSCSAVP00000017629.1 pep reftig:CSAV2.0:reftig_48:1427413:1432370:-1 gene:ENSCSAVG00000010377.1 transcript:ENSCSAVT00000017821.1**
Ciona|2105	>ENSCSAVP00000002105.1 pep reftig:CSAV2.0:reftig_91:1264886:1277611:1 gene:ENSCSAVG00000001240.1 transcript:ENSCSAVT00000002142.1
Ciona|2106	>ENSCSAVP00000002106.1 pep reftig:CSAV2.0:reftig_91:1264892:1276496:1 gene:ENSCSAVG00000001240.1 transcript:ENSCSAVT00000002143.1
**Ciona|2107**	**>ENSCSAVP00000002107.1 pep reftig:CSAV2.0:reftig_91:1264892:1270976:1 gene:ENSCSAVG00000001240.1 transcript:ENSCSAVT00000002144.1**
Ciona|2108	>ENSCSAVP00000002108.1 pep reftig:CSAV2.0:reftig_91:1265478:1277275:1 gene:ENSCSAVG00000001240.1 transcript:ENSCSAVT00000002145.1
Ciona|2109	>ENSCSAVP00000002109.1 pep reftig:CSAV2.0:reftig_91:1265490:1277435:1 gene:ENSCSAVG00000001240.1 transcript:ENSCSAVT00000002146.1
Ciona|4382	>ENSCSAVP00000004383.1 pep reftig:CSAV2.0:reftig_1:291989:295218:1 gene:ENSCSAVG00000002592.1 transcript:ENSCSAVT00000004447.1
**Ciona|899**	**>ENSCSAVP00000000899.1 pep reftig:CSAV2.0:reftig_209:145478:149484:1 gene:ENSCSAVG00000000511.1 transcript:ENSCSAVT00000000909.1**
Ciona|900	>ENSCSAVP00000000900.1 pep reftig:CSAV2.0:reftig_209:147742:157205:1 gene:ENSCSAVG00000000511.1 transcript:ENSCSAVT00000000910.1
Ciona|901	>ENSCSAVP00000000901.1 pep reftig:CSAV2.0:reftig_209:147751:157192:1 gene:ENSCSAVG00000000511.1 transcript:ENSCSAVT00000000911.1
Ciona|902	>ENSCSAVP00000000902.1 pep reftig:CSAV2.0:reftig_209:147835:157232:1 gene:ENSCSAVG00000000511.1 transcript:ENSCSAVT00000000912.1
Ciona|9289	>ENSCSAVP00000009293.1 pep reftig:CSAV2.0:reftig_88:299788:331188:-1 gene:ENSCSAVG00000005480.1 transcript:ENSCSAVT00000009410.1
Ciona|9290	>ENSCSAVP00000009294.1 pep reftig:CSAV2.0:reftig_88:299821:334624:-1 gene:ENSCSAVG00000005480.1 transcript:ENSCSAVT00000009411.1
Danio|11498	XP_700447.5
**Danio|11500**	**XP_021332325.1**
Danio|13137	XP_021332832.1
Danio|15182	XP_691883.4
Danio|16224	XP_005166620.1
Danio|18753	XP_689838.4
Danio|19227	XP_009302928.1
Danio|20031	XP_017213373.2
Danio|20240	XP_005167982.1
Danio|20241	XP_692892.6
Danio|22556	XP_021335380.1
Danio|22557	XP_009304092.1
Danio|22558	XP_021335381.1
Danio|22560	XP_021335382.1
Danio|24075	NP_957165.1
Danio|25723	XP_017214212.1
Danio|25726	NP_957396.2
Danio|2741	XP_009296519.1
**Danio|2743**	**XP_021324251.1**
Danio|28044	NP_001007161.1
Danio|29670	XP_692819.3

Homo|4290	NP_001032416.1
Homo|46939	NP_001229247.1
Homo|46940	XP_016869027.1
Homo|46942	NP_002594.1
Homo|56853	NP_006195.3
Homo|56854	XP_016871811.1
Homo|59908	NP_000913.2
Homo|59909	XP_016873400.1
Homo|62728	NP_002590.1
Homo|62732	XP_005274097.1
Homo|66037	NP_000912.3
Homo|66038	XP_016874909.1
Homo|67655	NP_000915.1
Homo|67656	XP_011536758.1
Homo|67657	NP_001159447.1
Homo|78789	NP_002596.1
Homo|78790	NP_775656.1
Homo|78791	XP_016877797.1
**Homo|78792**	**XP_016877799.1**
Homo|78796	XP_011519970.1
Homo|92889	NP_001230050.1
Homo|92890	NP_001104777.1
Homo|92893	NP_001104778.1
Homo|92894	NP_001104779.1
Homo|92895	NP_006193.1
Homo|93769	XP_016882355.1
Homo|93770	NP_001092288.1
**Ixodes|11817**	**>ISCW014308-PA**
**Ixodes|11818**	**>ISCW014309-PA**
Ixodes|16469	>ISCW020114-PA
**Ixodes|16470**	**>ISCW020115-PA**
Ixodes|18427	>ISCW022564-PA
Ixodes|18702	>ISCW022913-PA
Ixodes|19235	>ISCW023601-PA
Ixodes|4270	>ISCW005165-PA

Danio|30586	XP_021322214.1
Danio|31246	XP_697567.3
Danio|34331	NP_001074096.1
Danio|39864	XP_009293455.1
Danio|40340	XP_685002.1
Danio|4127	XP_017207862.1
Danio|4129	XP_003201807.3
Danio|45592	NP_001161735.1
Danio|45593	XP_005162727.1
Danio|45594	XP_009295653.1
Danio|45596	XP_005162725.1
Danio|45598	XP_021325775.1
Danio|45599	XP_005162729.1
Danio|456	XP_021331717.1
Danio|45600	XP_021325774.1
Danio|45601	XP_009295655.1
Danio|45880	XP_021326050.1
Danio|48662	XP_009296827.1
Danio|48663	XP_021322966.1
Danio|48664	XP_021322962.1
Danio|48666	XP_021326922.1
Danio|49395	XP_021332833.1
Danio|49396	XP_021327461.1
Danio|52241	XP_021329820.1
Danio|52242	XP_009302144.2
Danio|52243	XP_021329821.1
Danio|6356	XP_009298019.1
Danio|6357	XP_005164295.1
Danio|7020	XP_009298269.1
Daphnia|10625	>jgi|Dappu1|321218|NCBI_GNO_3900122
Daphnia|12707	>jgi|Dappu1|57148|e_gw1.61.4.1
Daphnia|1280	>jgi|Dappu1|311486|NCBI_GNO_0400260

Ixodes|5678	>ISCW006877-PA
Ixodes|9171	>ISCW011071-PA
Meloidogynefloridensis|13842	>genemark-nMf.1.1.scaf02732-processed-gene-0.2-mRNA-1 transcript
Meloidogynefloridensis|18998	>maker-nMf.1.1.scaf11742-augustus-gene-0.3-mRNA-1 transcript
**Meloidogynefloridensis|20716**	**>maker-nMf.1.1.scaf11625-snap-gene-0.6-mRNA-1 transcript**
Meloidogynefloridensis|2363	>maker-nMf.1.1.scaf00433-augustus-gene-0.19-mRNA-1 transcript
**Meloidogynefloridensis|25761**	**>maker-nMf.1.1.scaf01053-snap-gene-0.10-mRNA-1 transcript**
**Meloidogynefloridensis|2805**	**>genemark-nMf.1.1.scaf18942-processed-gene-0.1-mRNA-1 transcript**
**Meloidogynefloridensis|30127**	**>snap_masked-nMf.1.1.scaf16695-processed-gene-0.2-mRNA-1 transcript**
**Meloidogynefloridensis|45771**	**>snap_masked-nMf.1.1.scaf09731-processed-gene-0.4-mRNA-1 transcript**
Meloidogynefloridensis|46727	>maker-nMf.1.1.scaf00576-augustus-gene-0.17-mRNA-1 transcript
**Meloidogynefloridensis|49637**	**>maker-nMf.1.1.scaf04836-augustus-gene-0.6-mRNA-1 transcript**
Meloidogynefloridensis|5696	>maker-nMf.1.1.scaf00888-snap-gene-0.10-mRNA-1 transcript
Meloidogynehapla|13712	>MhA1_Contig894.frz3.gene3 transcript=MhA1_Contig894.frz3.gene3 gene=MhA1_Contig894.frz3.gene3
Meloidogynehapla|14035	>MhA1_Contig934.frz3.gene8 transcript=MhA1_Contig934.frz3.gene8 gene=MhA1_Contig934.frz3.gene8
Meloidogynehapla|4881	>MhA1_Contig1768.frz3.gene12 transcript=MhA1_Contig1768.frz3.gene12 gene=MhA1_Contig1768.frz3.gene12
**Meloidogynehapla|4882**	**>MhA1_Contig1768.frz3.gene13 transcript=MhA1_Contig1768.frz3.gene13 gene=MhA1_Contig1768.frz3.gene13**
**Meloidogynehapla|8164**	**>MhA1_Contig2771.frz3.fgene1 transcript=MhA1_Contig2771.frz3.fgene1 gene=MhA1_Contig2771.frz3.fgene1**
**Meloidogynehapla|8166**	**>MhA1_Contig2771.frz3.gene4 transcript=MhA1_Contig2771.frz3.gene4 gene=MhA1_Contig2771.frz3.gene4**
Meloidogynehapla|891	>MhA1_Contig111.frz3.gene11 transcript=MhA1_Contig111.frz3.gene11 gene=MhA1_Contig111.frz3.gene11
Meloidogynehapla|9145	>MhA1_Contig334.frz3.gene3 transcript=MhA1_Contig334.frz3.gene3 gene=MhA1_Contig334.frz3.gene3
**Meloidogyneincognita|3487**	**>Minc03277 transcript=Minc03277 gene=Minc03277**
**Meloidogyneincognita|3488**	**>Minc03278 transcript=Minc03278 gene=Minc03278**
Meloidogyneincognita|7615	>Minc07148a transcript=Minc07148a gene=Minc07148
Meloidogyneincognita|9561	>Minc09000a transcript=Minc09000a gene=Minc09000
Meloidogyneincognita|9738	>Minc09168a transcript=Minc09168a gene=Minc09168
Onchocerca|10576	>OVOC8433 wormpep=OVP05741 gene=WBGene00245242 locus=Ovo-pde-2 status=Predicted product="Putative 3 prime,5 prime-cyclic phosphodiesterase pde-2"
Onchocerca|3032	>OVOC1332 wormpep=OVP13035 gene=WBGene00238141 status=Predicted product="Putative 3 prime,5 prime-cyclic phosphodiesterase"
Onchocerca|3346	>OVOC1519 wormpep=OVP05268 gene=WBGene00238328 locus=Ovo-pde-5 status=Predicted product="Putative 3 prime,5 prime-cyclic phosphodiesterase pde-5"
Onchocerca|370	>OVOC10359 wormpep=OVP13519 gene=WBGene00247168 locus=Ovo-pde-4 status=Predicted product="Putative 3 prime,5 prime-cyclic phosphodiesterase pde-4"
Onchocerca|7198	>OVOC520 wormpep=OVP08661 gene=WBGene00237329 locus=Ovo-pde-1 status=Predicted product="Putative 3 prime,5 prime-cyclic phosphodiesterase"
Onchocerca|947	>OVOC10908 wormpep=OVP00944 gene=WBGene00247717 locus=Ovo-pde-3 status=Predicted product="Putative 3 prime,5 prime-cyclic phosphodiesterase pde-3"

Daphnia|13611	>jgi|Dappu1|321679|NCBI_GNO_4200057
**Daphnia|13612**	**>jgi|Dappu1|321680|NCBI_GNO_4200058**
Daphnia|14700	>jgi|Dappu1|101322|fgenesh1_pg.C_scaffold_17000273
Daphnia|19986	>jgi|Dappu1|59461|e_gw1.81.27.1
**Daphnia|24135**	**>jgi|Dappu1|62223|e_gw1.114.62.1**
Daphnia|4386	>jgi|Dappu1|303149|PASA_GEN_0200260
**Daphnia|5240**	**>jgi|Dappu1|55159|e_gw1.46.61.1**
Daphnia|5631	>jgi|Dappu1|63312|e_gw1.138.1.1
**Daphnia|7212**	**>jgi|Dappu1|250482|SNAP_00018540**
**Daphnia|7213**	**>jgi|Dappu1|306811|PASA_GEN_5300087**
Daphnia|892	>jgi|Dappu1|30651|gw1.5.536.1
Drosophila|11009	[Source:FlyBase;Acc:FBgn0266377]
Drosophila|11011	[Source:FlyBase;Acc:FBgn0266377]
Drosophila|11014	[Source:FlyBase;Acc:FBgn0266377]
Drosophila|17892	[Source:FlyBase;Acc:FBgn0085370]
Drosophila|24282	[Source:FlyBase;Acc:FBgn0038237]
Drosophila|6637	[Source:FlyBase;Acc:FBgn0000479]
Drosophila|6638	[Source:FlyBase;Acc:FBgn0000479]
Drosophila|6642	[Source:FlyBase;Acc:FBgn0000479]
Drosophila|6644	[Source:FlyBase;Acc:FBgn0000479]
Drosophila|8783	[Source:UniProtKB/TrEMBL;Acc:M9PAZ8]
Drosophila|8784	[Source:UniProtKB/TrEMBL;Acc:M9PAZ8]
Drosophila|8785	[Source:UniProtKB/TrEMBL;Acc:B7YZV2]
Drosophila|8786	[Source:UniProtKB/TrEMBL;Acc:B7YZV2]
Drosophila|8787	[Source:UniProtKB/TrEMBL;Acc:B7YZV2]
Drosophila|8789	[Source:UniProtKB/TrEMBL;Acc:B7YZV2]

Pristionchus|11647	>PPA17037 wormpep=PP38823 gene=WBGene00106591 locus=Ppa-pde-6 status=Predicted
Pristionchus|15245	>PPA22326 wormpep=PP47738 gene=WBGene00111880 locus=Ppa-pde-3 status=Predicted
Pristionchus|16689	>PPA24409 wormpep=PP39995 gene=WBGene00113963 locus=Ppa-pde-1 status=Predicted
**Pristionchus|16690**	**>PPA24410 wormpep=PP31120 gene=WBGene00113964 status=Predicted**
**Pristionchus|18991**	**>PPA27676 wormpep=PP30757 gene=WBGene00117230 status=Predicted**
**Pristionchus|18998**	**>PPA27684 wormpep=PP16366 gene=WBGene00117238 status=Predicted**
Pristionchus|2021	>PPA02836 wormpep=PP48058 gene=WBGene00092390 locus=Ppa-pde-4 status=Predicted
**Pristionchus|2022**	**>PPA02837 wormpep=PP13544 gene=WBGene00092391 status=Predicted**
**Pristionchus|9068**	**>PPA13116 wormpep=PP35371 gene=WBGene00102670 status=Predicted**
Saccoglossus|10334	XP_002736042.1
Saccoglossus|10347	XP_006818123.1
Saccoglossus|12053	XP_006819270.1
Saccoglossus|13949	XP_006820514.1
Saccoglossus|17881	XP_006823275.1
**Saccoglossus|22061**	**XP_006826108.1**
Saccoglossus|4308	XP_006814032.1
Saccoglossus|6430	XP_002733933.2
Saccoglossus|7448	XP_006816133.1
**Saccoglossus|8045**	**XP_006816549.1**
Saccoglossus|9347	XP_006817445.1
Saccoglossus|9411	XP_006817485.1
Saccoglossus|9513	XP_006817553.1
Strongyloides|11253	uniprot=A0A090KWL9
Strongyloides|3977	uniprot=A0A090L4W1
Strongyloides|4812	uniprot=A0A090LDV4
Strongyloides|5717	uniprot=A0A090LEJ3
Strongyloides|7006	uniprot=A0A090LDH7
Strongyloides|9977	uniprot=A0A090LLU9
