# Supplementary material for: Pharmacological and molecular dynamics analyses of differences in inhibitor binding to human and nematode PDE4: Implications for management of parasitic nematodes
Source: PLoS One. 2019 Mar 27;14(3):e0214554. doi: 10.1371/journal.pone.0214554 (PMC6436744; doi:10.1371/journal.pone.0214554)
Supplement: S3 Table — The table lists the amino acids present in the human and C. elegans PDE4 catalytic domain at the given position, as well as the residue number for each of the four different protein sequences used. C. elegans residue begins at Asn285 and ends at Pro608 (Uniprot ID: S6FCW6). Blank cells denote no amino acid residue present at that position. (PDF) [file pone.0214554.s003.pdf]

S3 Table. Table of correspondence for the amino acid residue numbers of human PDE4D (PDB IDs 3G4L, 1MKD, and 1ZKN) and for the corresponding *C. elegans* PDE4 residues. The table lists the amino acids present in the human and *C. elegans* PDE4 catalytic domain at the given position, as well as the residue number for each of the four different protein sequences used. *C. elegans* residue begins at Asn285 and ends at Pro608 (Uniprot ID: S6FCW6). Blank cells denote no amino acid residue present at that position.

Pos.	human PDE4D a.a.	3G4L Pos.	1MKD Pos.	1ZKN Pos.	*C. elegans* PDE4 a.a.	*C. elegans* MD pos.
1	S	243				
2	S	244				
3	I	245		79		
4	P	246		80		
5	R	247		81		
6	F	248		82		
7	G	249		83		
8	V	250		84		
9	K	251		85		
10	T	252	183	86		
11	E	253	184	87	N	1
12	Q	254	185	88	C	2
13	E	255	186	89	A	3
14	D	256	187	90	R	4
15	V	257	188	91	E	5
16	L	258	189	92	I	6
17	A	259	190	93	A	7
18	K	260	191	94	V	8
19	E	261	192	95	H	9
20	L	262	193	96	M	10
21	E	263	194	97	Q	11
22	D	264	195	98	R	12
23	V	265	196	99	L	13
24	N	266	197	100	D	14
25	K	267	198	101	D	15
26	W	268	199	102	W	16
27	G	269	200	103	G	17
28	L	270	201	104	P	18
29	H	271	202	105	D	19
30	V	272	203	106	V	20
31	F	273	204	107	F	21
32	R	274	205	108	K	22
33	I	275	206	109	I	23
34	A	276	207	110	D	24
35	E	277	208	111	E	25
36	L	278	209	112	L	26
37	S	279	210	113	S	27
38	G	280	211	114	K	28
39	N	281	212	115	N	29
40	R	282	213	116	H	30
41	P	283	214	117	S	31
42	L	284	215	118	L	32
43	T	285	216	119	T	33
44	V	286	217	120	V	34
45	I	287	218	121	V	35
46	M	288	219	122	T	36
47	H	289	220	123	F	37
48	T	290	221	124	S	38
49	I	291	222	125	L	39
50	F	292	223	126	L	40
51	Q	293	224	127	R	41
52	E	294	225	128	Q	42
53	R	295	226	129	R	43
54	D	296	227	130	N	44
55	L	297	228	131	L	45
56	L	298	229	132	F	46
57	K	299	230	133	K	47
58	T	300	231	134	T	48
59	F	301	232	135	F	49
60	K	302	233	136	E	50
61	I	303	234	137	I	51
62	P	304	235	138	H	52
63	V	305	236	139	Q	53
64	D	306	237	140	S	54
65	T	307	238	141	T	55
66	L	308	239	142	L	56
67	I	309	240	143	V	57
68	T	310	241	144	T	58
69	Y	311	242	145	Y	59
70	L	312	243	146	L	60
71	M	313	244	147	L	61
72	T	314	245	148	N	62
73	L	315	246	149	L	63
74	E	316	247	150	E	64
75	D	317	248	151	H	65
76	H	318	249	152	H	66
77	Y	319	250	153	Y	67
78	H	320	251	154	R	68
79	A	321	252	155	N	69
80	D	322	253	156	N	70
81	V	323	254	157		
82	A	324	255	158	H	71
83	Y	325	256	159	Y	72
84	H	326	257	160	H	73

Pos.	human PDE4D a.a.	3G4L Pos.	1MKD Pos.	1ZKN Pos.	*C. elegans* PDE4 a.a.	*C. elegans* MD pos.
85	N	327	258	161	N	74
86	N	328	259	162	F	75
87	I	329	260	163	I	76
88	H	330	261	164	H	77
89	A	331	262	165	A	78
90	A	332	263	166	A	79
91	D	333	264	167	D	80
92	V	334	265	168	V	81
93	V	335	266	169	A	82
94	Q	336	267	170	Q	83
95	S	337	268	171	S	84
96	T	338	269	172	M	85
97	H	339	270	173	H	86
98	V	340	271	174	V	87
99	L	341	272	175	L	88
100	L	342	273	176	L	89
101	S	343	274	177	M	90
102	T	344	275	178	S	91
103	P	345	276	179	P	92
104	A	346	277	180	V	93
105	L	347	278	181	L	94
106	E	348	279	182	T	95
107	A	349	280	183	E	96
108	V	350	281	184	V	97
109	F	351	282	185	F	98
110	T	352	283	186	T	99
111	D	353	284	187	D	100
112	L	354	285	188	L	101
113	E	355	286	189	E	102
114	I	356	287	190	V	103
115	L	357	288	191	L	104
116	A	358	289	192	A	105
117	A	359	290	193	A	106
118	I	360	291	194	I	107
119	F	361	292	195	F	108
120	A	362	293	196	A	109
121	S	363	294	197	G	110
122	A	364	295	198	A	111
123	I	365	296	199	V	112
124	H	366	297	200	H	113
125	D	367	298	201	D	114
126	V	368	299	202	V	115

Pos.	human PDE4D a.a.	3G4L Pos.	1MKD Pos.	1ZKN Pos.	*C. elegans* PDE4 a.a.	*C. elegans* MD pos.
127	D	369	300	203	D	116
128	H	370	301	204	H	117
129	P	371	302	205	P	118
130	G	372	303	206	G	119
131	V	373	304	207	F	120
132	S	374	305	208	T	121
133	N	375	306	209	N	122
134	Q	376	307	210	Q	123
135	F	377	308	211	Y	124
136	L	378	309	212	L	125
137	I	379	310	213	I	126
138	N	380	311	214	N	127
139	T	381	312	215	S	128
140	N	382	313	216	N	129
141	S	383	314	217	N	130
142	E	384	315	218	E	131
143	L	385	316	219	L	132
144	A	386	317	220	A	133
145	L	387	318	221	I	134
146	M	388	319	222	M	135
147	Y	389	320	223	Y	136
148	N	390	321	224	N	137
149	D	391	322	225	D	138
150	S	392	323	226	E	139
151	S	393	324	227	S	140
152	V	394	325	228	V	141
153	L	395	326	229	L	142
154	E	396	327	230	E	143
155	N	397	328	231	Q	144
156	H	398	329	232	H	145
157	H	399	330	233	H	146
158	L	400	331	234	L	147
159	A	401	332	235	A	148
160	V	402	333	236	V	149
161	G	403	334	237	A	150
162	F	404	335	238	F	151
163	K	405	336	239	K	152
164	L	406	337	240	L	153
165	L	407	338	241	L	154
166	Q	408	339	242	Q	155
167	E	409	340	243	D	156
168	E	410	341	244	S	157

Pos.	human PDE4D a.a.	3G4L Pos.	1MKD Pos.	1ZKN Pos.	*C. elegans* PDE4 a.a.	*C. elegans* MD pos.
169	N	411	342	245	N	158
170	C	412	343	246	C	159
171	D	413	344	247	D	160
172	I	414	345	248	F	161
173	F	415	346	249	L	162
174	Q	416	347	250	A	163
175	N	417	348	251	N	164
176	L	418	349	252	L	165
177	T	419	350	253	S	166
178	K	420	351	254	R	167
179	K	421	352	255	K	168
180	Q	422	353	256	Q	169
181	R	423	354	257	R	170
182	Q	424	355	258	L	171
183	S	425	356	259	Q	172
184	L	426	357	260	F	173
185	R	427	358	261	R	174
186	K	428	359	262	K	175
187	M	429	360	263	I	176
188	V	430	361	264	V	177
189	I	431	362	265	I	178
190	D	432	363	266	D	179
191	I	433	364	267	M	180
192	V	434	365	268	V	181
193	L	435	366	269	L	182
194	A	436	367	270	A	183
195	T	437	368	271	T	184
196	D	438	369	272	D	185
197	M	439	370	273	M	186
198	S	440	371	274	S	187
199	K	441	372	275	K	188
200	H	442	373	276	H	189
201	M	443	374	277	M	190
202	N	444	375	278	S	191
203	L	445	376	279	L	192
204	L	446	377	280	L	193
205	A	447	378	281	A	194
206	D	448	379	282	D	195
207	L	449	380	283	L	196
208	K	450	381	284	K	197
209	T	451	382	285	T	198
210	M	452	383	286	M	199

Pos.	human PDE4D a.a.	3G4L Pos.	1MKD Pos.	1ZKN Pos.	*C. elegans* PDE4 a.a.	*C. elegans* MD pos.
211	V	453	384	287	V	200
212	E	454	385	288	E	201
213	T	455	386	289	A	202
214	K	456	387	290	K	203
215	K	457	388	291	K	204
216	V	458	389	292	V	205
217	T	459	390	293	A	206
218	S	460	391	294	G	207
219	S	461	392	295	N	208
220	G	462	393	296	N	209
221	V	463	394	297	V	210
222	L	464	395	298	I	211
223	L	465	396	299	V	212
224	L	466	397	300	L	213
225	D	467	398	301	D	214
226	N	468	399	302	K	215
227	Y	469	400	303	Y	216
228	S	470	401	304	N	217
229	D	471	402	305	D	218
230	R	472	403	306	K	219
231	I	473	404	307	I	220
232	Q	474	405	308	Q	221
233	V	475	406	309	V	222
234	L	476	407	310	L	223
235	Q	477	408	311	Q	224
236	N	478	409	312	S	225
237	M	479	410	313	M	226
238	V	480	411	314	I	227
239	H	481	412	315	H	228
240	C	482	413	316	L	229
241	A	483	414	317	A	230
242	D	484	415	318	D	231
243	L	485	416	319	L	232
244	S	486	417	320	S	233
245	N	487	418	321	N	234
246	P	488	419	322	P	235
247	T	489	420	323	T	236
248	K	490	421	324	K	237
249	P	491	422	325	P	238
250	L	492	423	326	I	239
251	Q	493	424	327	E	240
252	L	494	425	328	L	241

Pos.	human PDE4D a.a.	3G4L Pos.	1MKD Pos.	1ZKN Pos.	*C. elegans* PDE4 a.a.	*C. elegans* MD pos.
253	Y	495	426	329	Y	242
254	R	496	427	330	Q	243
255	Q	497	428	331	Q	244
256	W	498	429	332	W	245
257	T	499	430	333	N	246
258	D	500	431	334	Q	247
259	R	501	432	335	R	248
260	I	502	433	336	I	249
261	M	503	434	337	M	250
262	E	504	435	338	E	251
263	E	505	436	339	E	252
264	F	506	437	340	Y	253
265	F	507	438	341	W	254
266	R	508	439	342	R	255
267	Q	509	440	343	Q	256
268	G	510	441	344	G	257
269	D	511	442	345	D	258
270	R	512	443	346	K	259
271	E	513	444	347	E	260
272	R	514	445	348	K	261
273	E	515	446	349	E	262
274	R	516	447	350	L	263
275	G	517	448	351	G	264
276	M	518	449	352	L	265
277	E	519	450	353	E	266
278	I	520	451	354	I	267
279	S	521	452	355	S	268
280	P	522	453	356	P	269
281	M	523	454	357	M	270
282	C	524	455	358	C	271
283	D	525	456	359	D	272
284	K	526	457	360	R	273
285	H	527	458	361	G	274
286	N	528	459	362	N	275
287	A	529	460	363	V	276
288	S	530	461	364	T	277
289	V	531	462	365	I	278
290	E	532	463	366	E	279
291	K	533	464	367	K	280
292	S	534	465	368	S	281
293	Q	535	466	369	Q	282
294	V	536	467	370	V	283

Pos.	human PDE4D a.a.	3G4L Pos.	1MKD Pos.	1ZKN Pos.	*C. elegans* PDE4 a.a.	*C. elegans* MD pos.
295	G	537	468	371	G	284
296	F	538	469	372	F	285
297	I	539	470	373	I	286
298	D	540	471	374	D	287
299	Y	541	472	375	Y	288
300	I	542	473	376	I	289
301	V	543	474	377	V	290
302	H	544	475	378	H	291
303	P	545	476	379	P	292
304	L	546	477	380	L	293
305	W	547	478	381	Y	294
306	E	548	479	382	E	295
307	T	549	480	383	T	296
308	W	550	481	384	W	297
309	A	551	482	385	A	298
310	D	552	483	386	D	299
311	L	553	484	387	L	300
312	V	554	485	388	V	301
313	H	555	486	389	Y	302
314	P	556	487	390	P	303
315	D	557	488	391	D	304
316	A	558	489	392	A	305
317	Q	559	490	393	Q	306
318	D	560	491	394	N	307
319	I	561	492	395	I	308
320	L	562	493	396	L	309
321	D	563	494	397	D	310
322	T	564	495	398	Q	311
323	L	565	496	399	L	312
324	E	566	497	400	E	313
325	D	567	498	401	E	314
326	N	568	499	402	N	315
327	R	569	500	403	R	316
328	E	570	501	404	E	317
329	W	571	502	405	W	318
330	Y	572	503	406	Y	319
331	Q	573	504	407	Q	320
332	S	574	505	408	S	321
333	T	575	506	409	R	322
334	I	576	507	410	I	323
335	P	577	508	411	P	324
336	Q		509	412		
337	S		510			
