# Supplementary material for: Pharmacological and molecular dynamics analyses of differences in inhibitor binding to human and nematode PDE4: Implications for management of parasitic nematodes
Source: PLoS One. 2019 Mar 27;14(3):e0214554. doi: 10.1371/journal.pone.0214554 (PMC6436744; doi:10.1371/journal.pone.0214554)
Supplement: S4 Table — (PDF) [file pone.0214554.s004.pdf]

S4 Table. Details of MD simulations.

Run No.	System	Initial structure	Run length (ns)	Ligand	System size (atoms)	No. of runs
1	PDE4D	1ZKN	120	IBMX	55798	2
2	PDE4D	3G4L	120	roflumilast	56407	2
3	PDE4D	1MKD	120	zardaverine	60529	2
4	*C.elegans* PDE4	homology	120	IBMX	58304	2
5	*C.elegans* PDE4	homology	120	roflumilast	56407	2
6	*C.elegans* PDE4	homology	120	zardaverine	60529	2
7	PDE4D	1ZKN, 3G4L, 1MKD	120	-	64563<br>58980<br>57499	3
8	*C.elegans* PDE4	homology	120	-	67009<br>62234<br>65484	3
