# Supplementary material for: Pharmacological and molecular dynamics analyses of differences in inhibitor binding to human and nematode PDE4: Implications for management of parasitic nematodes
Source: PLoS One. 2019 Mar 27;14(3):e0214554. doi: 10.1371/journal.pone.0214554 (PMC6436744; doi:10.1371/journal.pone.0214554)

S1 Figure. Phylogeny of the catalytic domain of putative PDE genes. Bootstrap analysis was run 100 times and any support values less than 50 were removed from the tree for clarity.

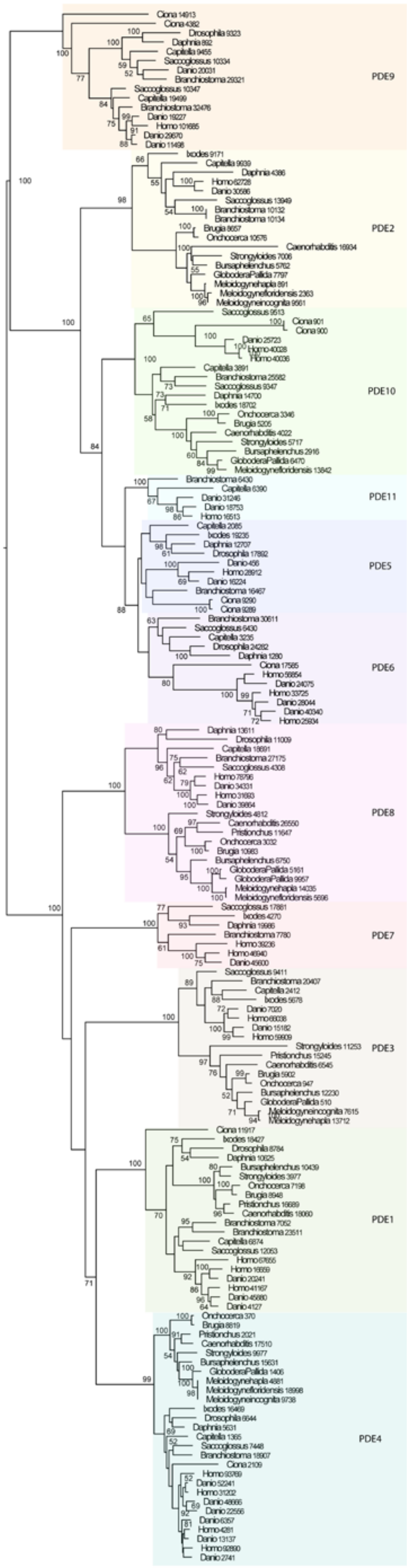

Supplement: S1 Fig — Bootstrap analysis was run 100 times and any support values less than 50 were removed from the tree for clarity. (PDF) [file pone.0214554.s005.pdf]
