# Supplementary material for: Pharmacological and molecular dynamics analyses of differences in inhibitor binding to human and nematode PDE4: Implications for management of parasitic nematodes
Source: PLoS One. 2019 Mar 27;14(3):e0214554. doi: 10.1371/journal.pone.0214554 (PMC6436744; doi:10.1371/journal.pone.0214554)

S2 Figure. The traces of root-mean-squared-deviation (RMSD) vs. simulation time (ns) for PDE4D and *C. elegans* PDE4. (a and b) Two independent simulation runs for complexes of human PDE4D and *C. elegans* PDE4 with IBMX, zardaverine, or roflumilast. (c) RMSD traces of three independent simulation runs of apo-PDE4D and apo-*C. elegans* PDE4.

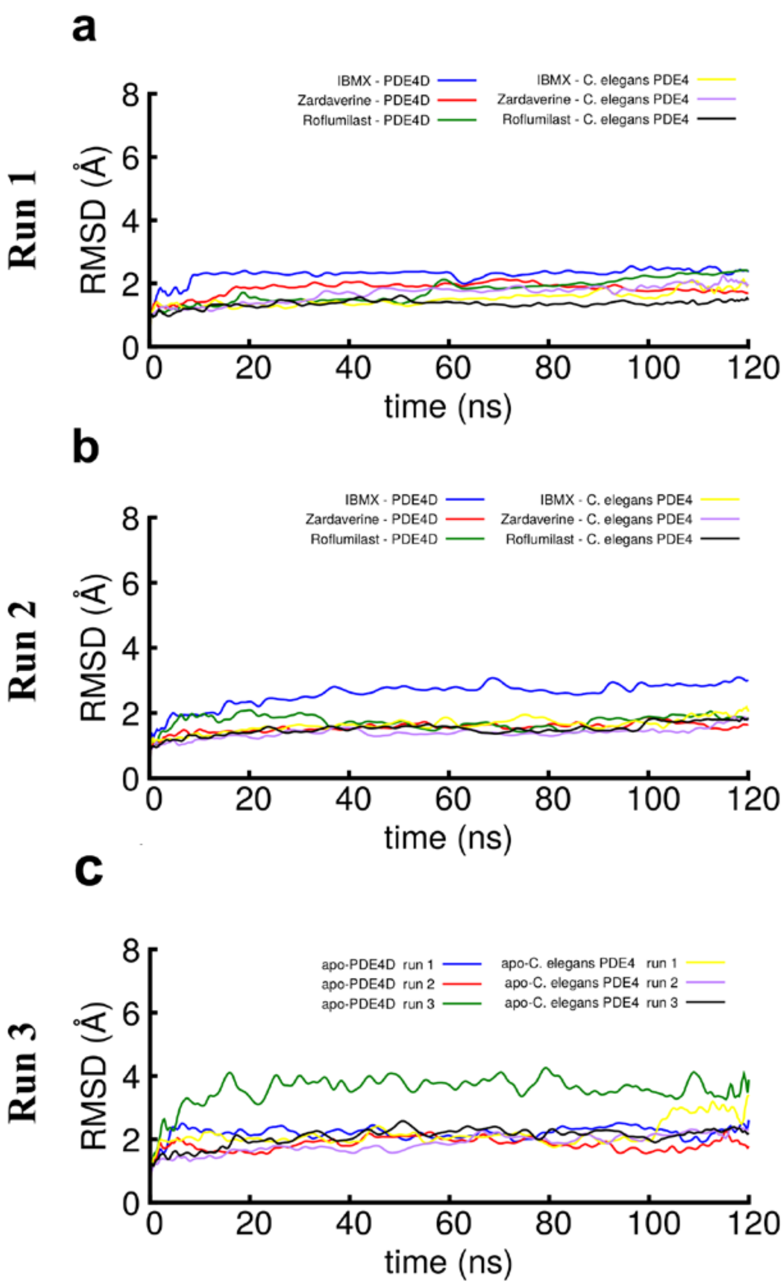

Supplement: S2 Fig — The traces of root-mean-squared-deviation (RMSD) vs. simulation time (ns) for PDE4D and C. elegans PDE4. (a and b) Two independent simulation runs for complexes of human PDE4D and C. elegans PDE4 with IBMX, zardaverine, or roflumilast. (c) RMSD traces of three independent simulation runs of apo-PDE4D and apo-C. elegans PDE4. (PDF) [file pone.0214554.s006.pdf]
