# Supplementary material for: Pharmacological and molecular dynamics analyses of differences in inhibitor binding to human and nematode PDE4: Implications for management of parasitic nematodes
Source: PLoS One. 2019 Mar 27;14(3):e0214554. doi: 10.1371/journal.pone.0214554 (PMC6436744; doi:10.1371/journal.pone.0214554)

S3 Figure. The nonbonded interaction energy analysis between residues in the inhibitor binding pocket of PDE4D and *C. elegans* PDE4 for the first simulation run. See Fig. 3 and Fig. 4a and 4b for depictions of the binding pocket and the 32 residues analyzed with bound (a) IBMX, (b) zardaverine, and (c) roflumilast. Amino acid residues in blue text denote residues that differ between human and *C. elegans* PDE4 sequences.

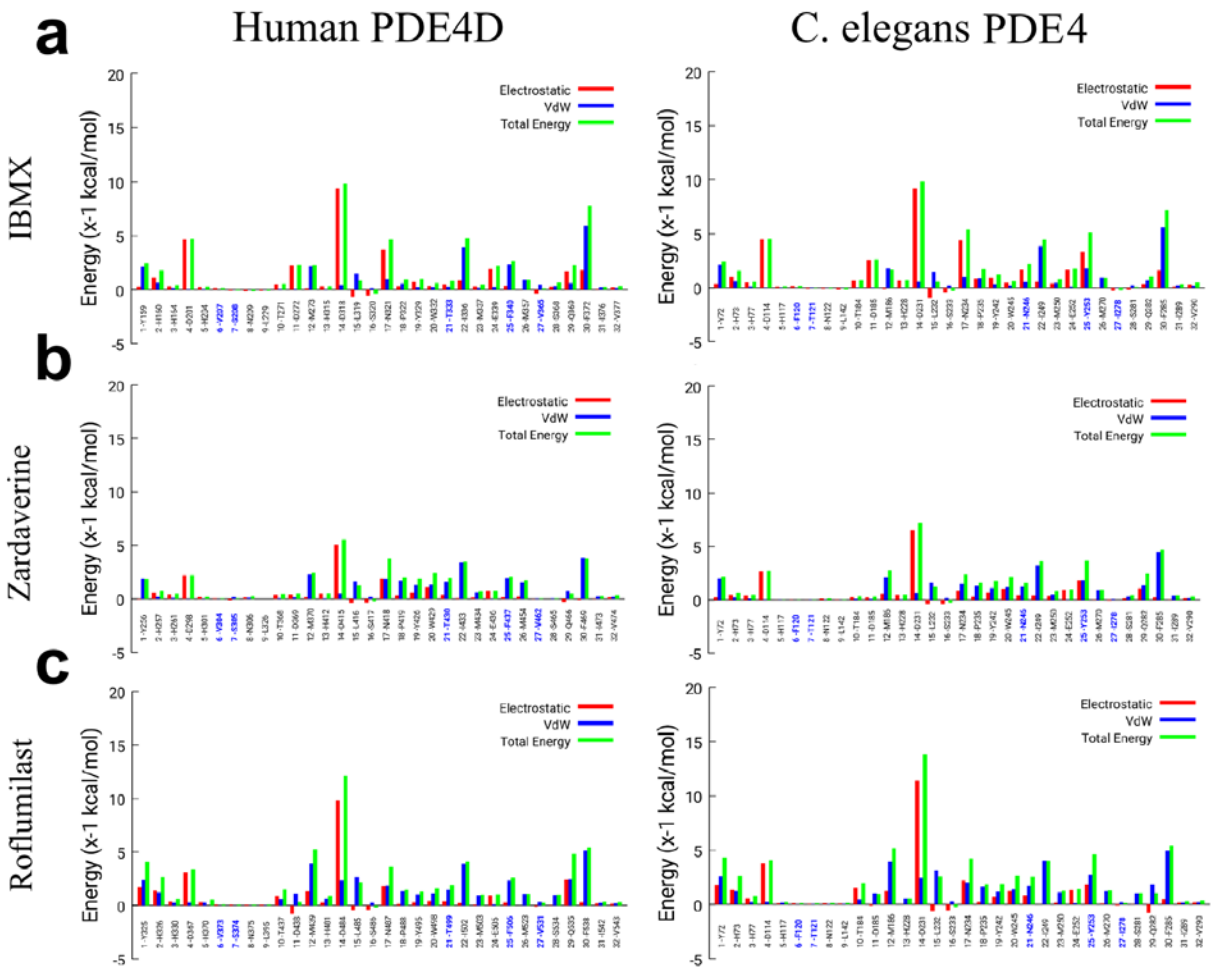

Supplement: S3 Fig — See Fig 3 and Fig 4A and 4B for depictions of the binding pocket and the 32 residues analyzed with bound (a) IBMX, (b) zardaverine, and (c) roflumilast. Amino acid residues in blue text denote residues that differ between human and C. elegans PDE4 sequences. (PDF) [file pone.0214554.s007.pdf]
