# Supplementary material for: Pharmacological and molecular dynamics analyses of differences in inhibitor binding to human and nematode PDE4: Implications for management of parasitic nematodes
Source: PLoS One. 2019 Mar 27;14(3):e0214554. doi: 10.1371/journal.pone.0214554 (PMC6436744; doi:10.1371/journal.pone.0214554)

S4 Figure. The nonbonded interaction energy analysis between residues in the inhibitor binding pocket of PDE4D and *C. elegans* PDE4 for the second simulation run. (a) IBMX, (b) zardaverine, and (c) roflumilast. Amino acid residues in blue text denote residues that differ between human and *C. elegans* PDE4.

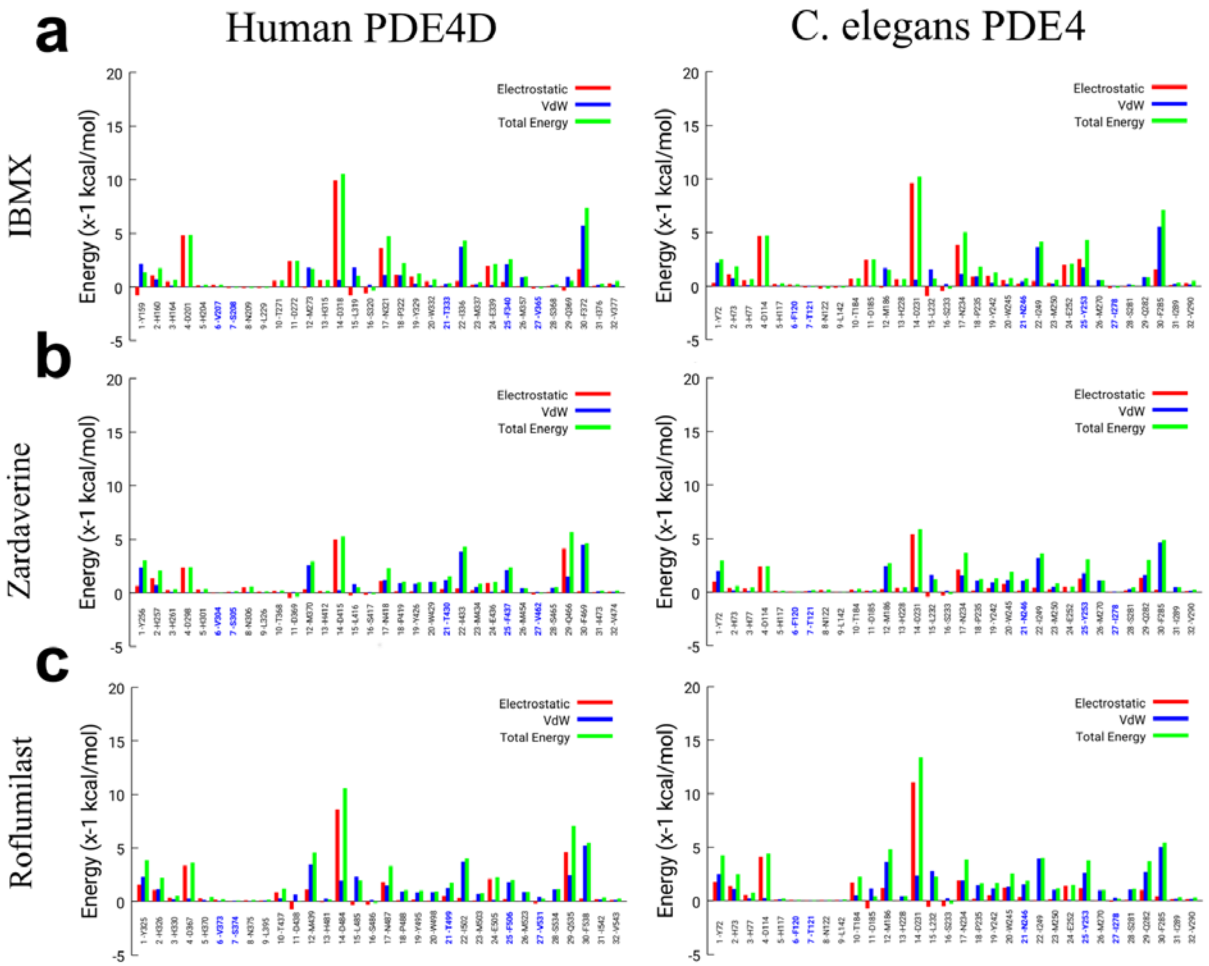

Supplement: S4 Fig — (a) IBMX, (b) zardaverine, and (c) roflumilast. Amino acid residues in blue text denote residues that differ between human and C. elegans PDE4. (PDF) [file pone.0214554.s008.pdf]
