# Supplementary material for: Pharmacological and molecular dynamics analyses of differences in inhibitor binding to human and nematode PDE4: Implications for management of parasitic nematodes
Source: PLoS One. 2019 Mar 27;14(3):e0214554. doi: 10.1371/journal.pone.0214554 (PMC6436744; doi:10.1371/journal.pone.0214554)

S6 Figure. Interatomic distances between C4 atom of F506(human)/O atom on the side chain of Y253(*C. elegans*) (blue dashed line, labeled 1) or the N_{δ} atom of Q369/Q282 (red dashed line, labeled 2) and the O4 oxygen of zardaverine bound to human PDE4D or *C. elegans* PDE4 obtained from two independent MD simulation runs, (a) run 1 and (b) run 2.

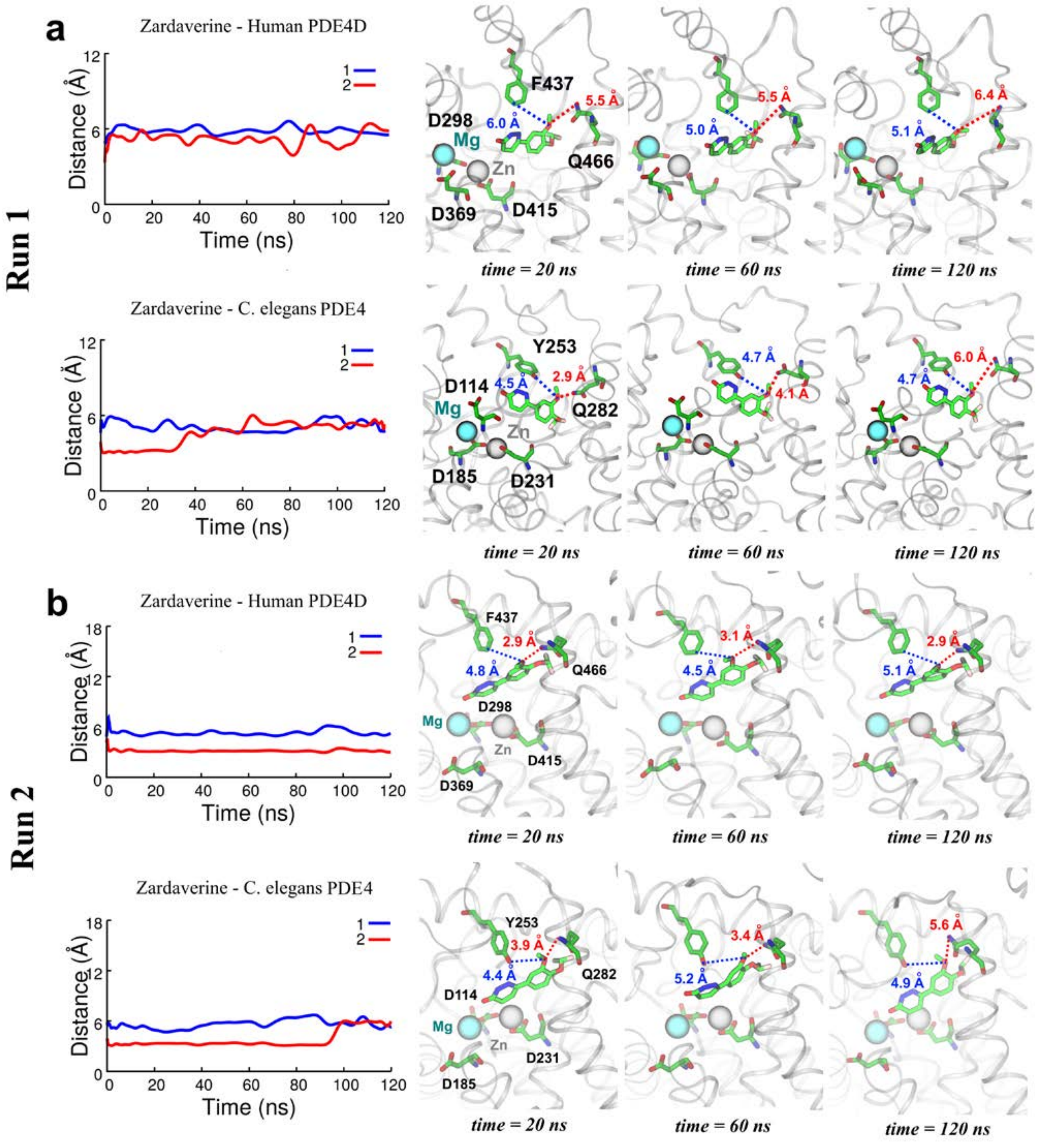

Supplement: S6 Fig — Interatomic distances between C4 atom of F506(human)/O atom on the side chain of Y253(C. elegans) (blue dashed line, labeled 1) or the Nδ atom of Q369/Q282 (red dashed line, labeled 2) and the O4 oxygen of zardaverine bound to human PDE4D or C. elegans PDE4 obtained from two independent MD simulation runs, (a) run 1 and (b) run 2. (PDF) [file pone.0214554.s010.pdf]
