# Supplementary material for: Pharmacological and molecular dynamics analyses of differences in inhibitor binding to human and nematode PDE4: Implications for management of parasitic nematodes
Source: PLoS One. 2019 Mar 27;14(3):e0214554. doi: 10.1371/journal.pone.0214554 (PMC6436744; doi:10.1371/journal.pone.0214554)

S8 Figure. Dynamic cross correlation matrices calculated for the C_α atoms of human PDE4D and *C. elegans* PDE4 complexed with IBMX (a), zardaverine (b), and roflumilast (c). Residues in the α14 and α15 helices are shown by areas between dashed-lines and solid-lines, respectively. Red tick-marks on the axes represent the 32 residues in the binding site (as depicted in Fig. 4a-b). The color scheme ranges from anticorrelation (-1.0, blue), no correlation (0, green), and positive correlation (+1.0, red). Values are the average for the two independent simulation runs.

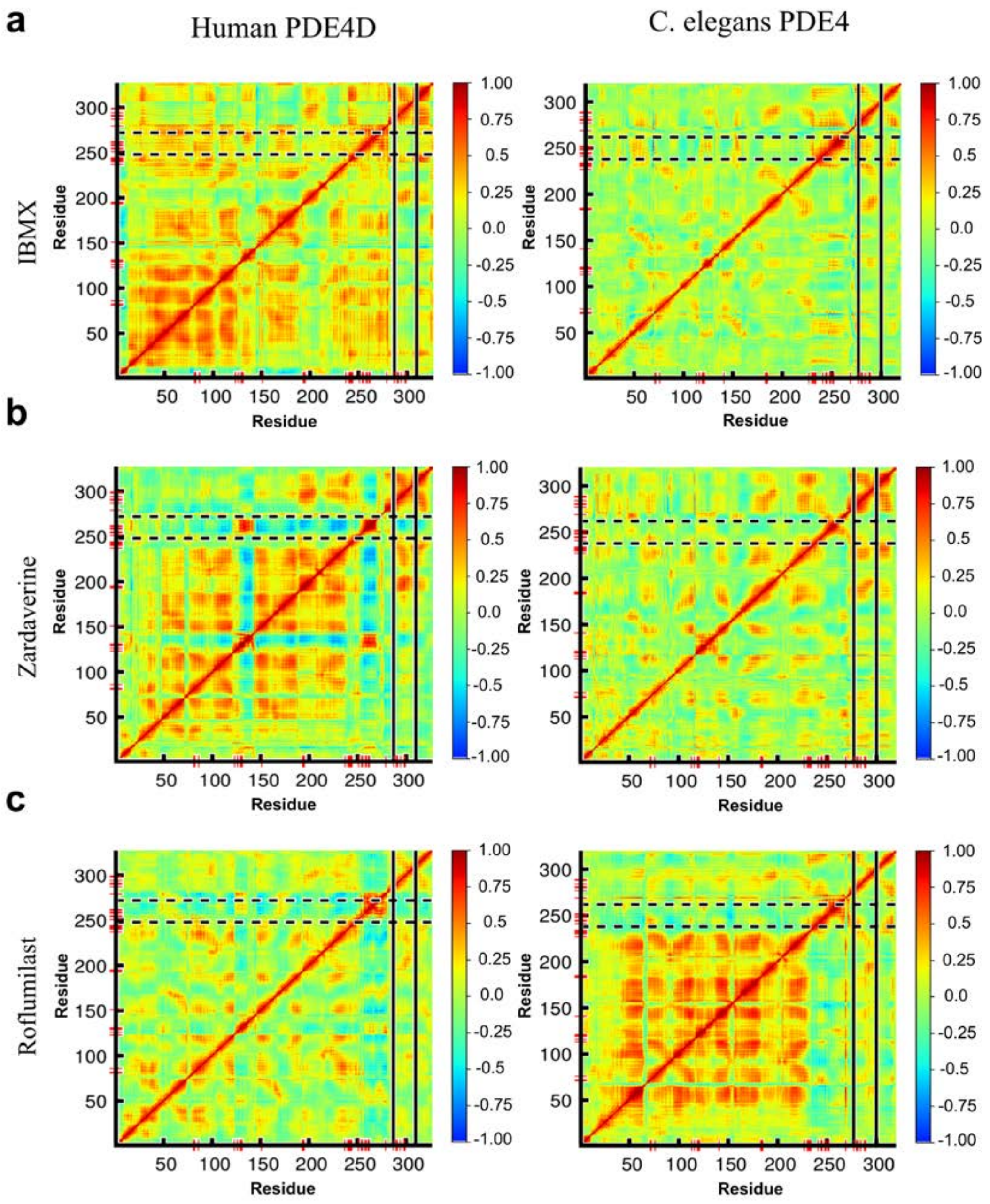

Supplement: S8 Fig — Dynamic cross correlation matrices calculated for the Cα atoms of human PDE4D and C. elegans PDE4 complexed with IBMX (a), zardaverine (b), and roflumilast (c). Residues in the α14 and α15 helices are shown by areas between dashed-lines and solid-lines, respectively. Red tick-marks on the axes represent the 32 residues in the binding site (as depicted in Fig 4A and 4B). The color scheme ranges from anticorrelation (-1.0, blue), no correlation (0, green), and positive correlation (+1.0, red). Values are the average for the two independent simulation runs. (PDF) [file pone.0214554.s012.pdf]
