# Supplementary material for: Pharmacological and molecular dynamics analyses of differences in inhibitor binding to human and nematode PDE4: Implications for management of parasitic nematodes
Source: PLoS One. 2019 Mar 27;14(3):e0214554. doi: 10.1371/journal.pone.0214554 (PMC6436744; doi:10.1371/journal.pone.0214554)

S9 Figure. Dynamic cross correlation matrices calculated for the C_α atoms of human PDE4D and *C. elegans* PDE4 in their apo state. Color scheme is the same as for Fig. S8. Panels a-c represent three independent simulations.

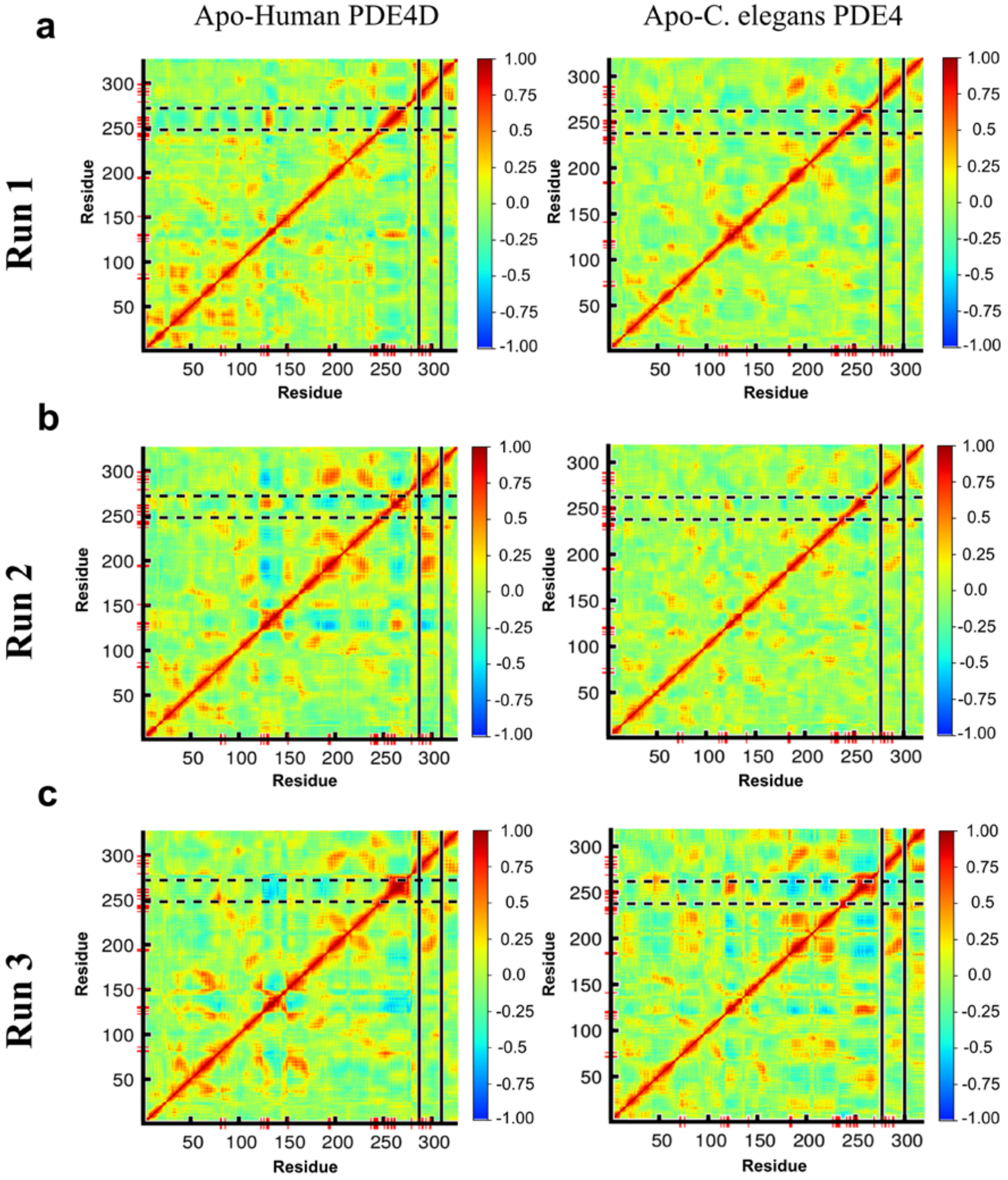

Supplement: S9 Fig — Color scheme is the same as for S8 Fig. Panels a-c represent three independent simulations. (PDF) [file pone.0214554.s013.pdf]
