# Supplementary material for: Pharmacological and molecular dynamics analyses of differences in inhibitor binding to human and nematode PDE4: Implications for management of parasitic nematodes
Source: PLoS One. 2019 Mar 27;14(3):e0214554. doi: 10.1371/journal.pone.0214554 (PMC6436744; doi:10.1371/journal.pone.0214554)

S10 Figure. Key salt-bridging interactions are shown based upon the first set of MD simulations of human PDE4D and *C. elegans* PDE4 with IBMX (a), zardaverine (b), and roflumilast (c). Three conserved salt-bridges are labeled in blue.

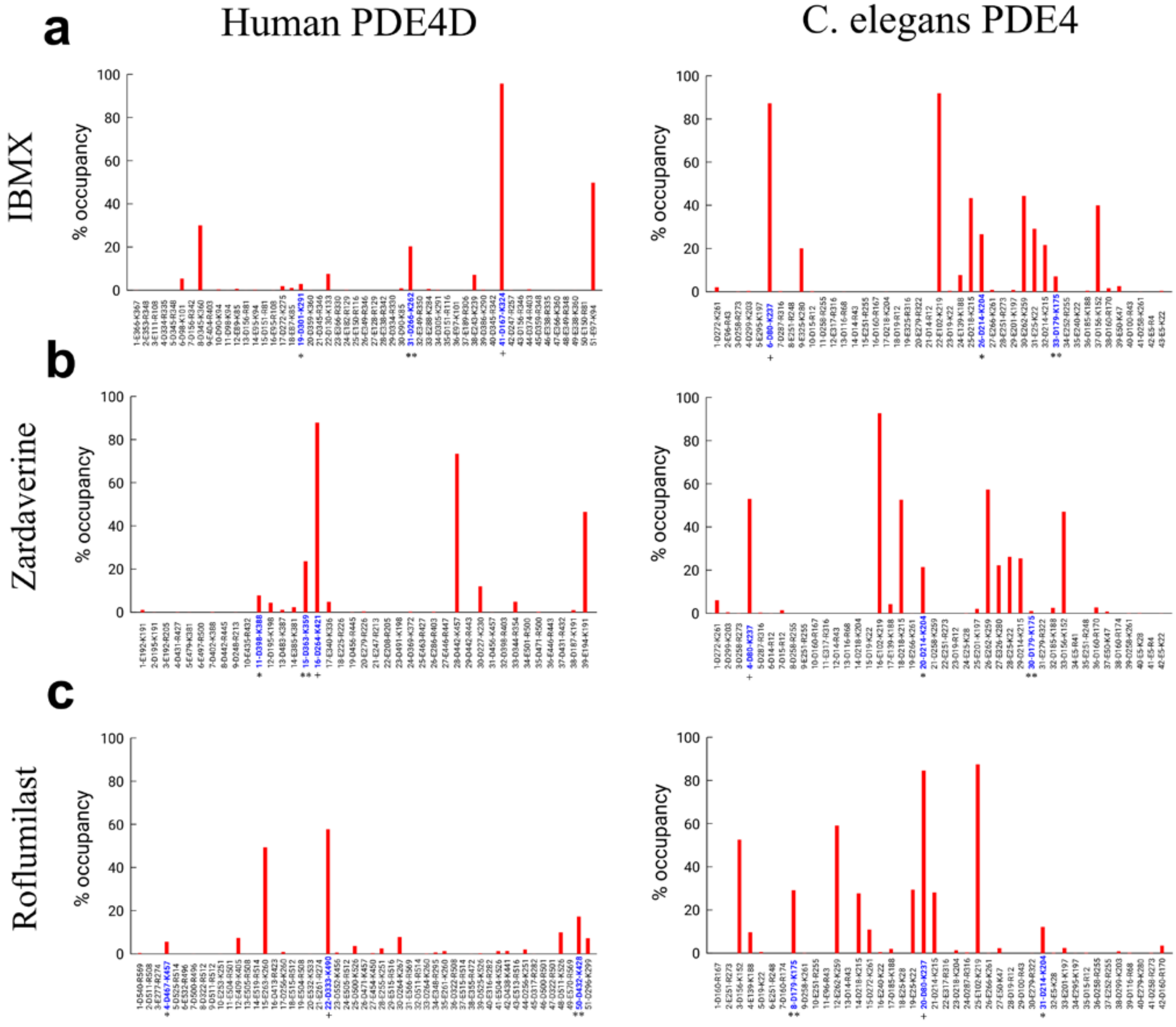

Supplement: S10 Fig — Key salt-bridging interactions are shown based upon the first set of MD simulations of human PDE4D and C. elegans PDE4 with IBMX (a), zardaverine (b), and roflumilast (c). Three conserved salt-bridges are labeled in blue. (PDF) [file pone.0214554.s014.pdf]
