# Supplementary material for: Pharmacological and molecular dynamics analyses of differences in inhibitor binding to human and nematode PDE4: Implications for management of parasitic nematodes
Source: PLoS One. 2019 Mar 27;14(3):e0214554. doi: 10.1371/journal.pone.0214554 (PMC6436744; doi:10.1371/journal.pone.0214554)

S11 Figure. Data similar to Fig. S10 are shown for a second set of MD simulations with the three inhibitors.

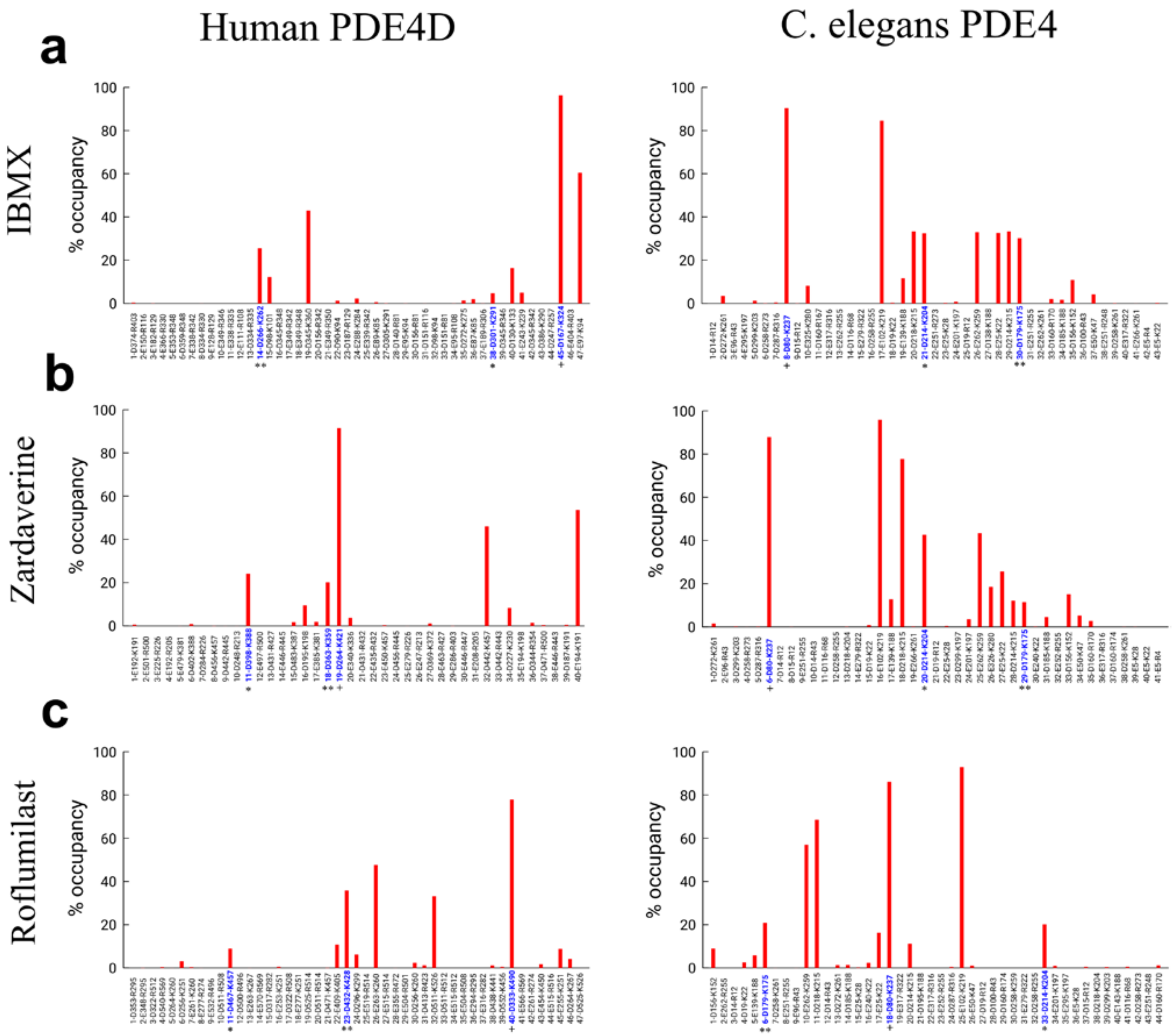

Supplement: S11 Fig — (PDF) [file pone.0214554.s015.pdf]
