# Supplementary material for: Pharmacological and molecular dynamics analyses of differences in inhibitor binding to human and nematode PDE4: Implications for management of parasitic nematodes
Source: PLoS One. 2019 Mar 27;14(3):e0214554. doi: 10.1371/journal.pone.0214554 (PMC6436744; doi:10.1371/journal.pone.0214554)

S12 Figure. Data similar to Fig. S10 are shown for three independent sets of MD simulations of apo human PDE4D and apo *C. elegans* PDE4.

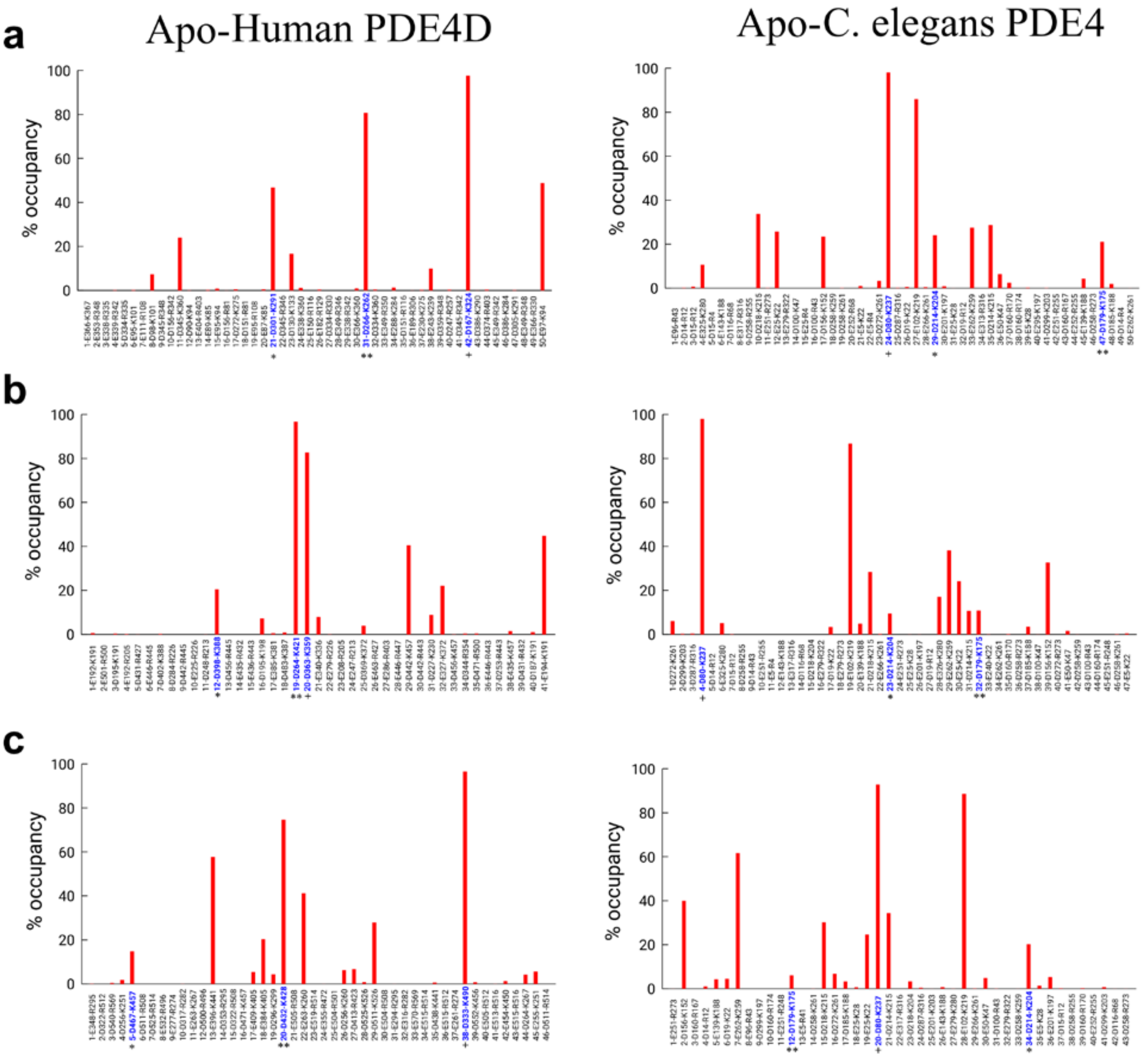

Supplement: S12 Fig — (PDF) [file pone.0214554.s016.pdf]
