# Supplementary material for: Pharmacological and molecular dynamics analyses of differences in inhibitor binding to human and nematode PDE4: Implications for management of parasitic nematodes
Source: PLoS One. 2019 Mar 27;14(3):e0214554. doi: 10.1371/journal.pone.0214554 (PMC6436744; doi:10.1371/journal.pone.0214554)

S13 Figure. *C. elegans* PDE4 catalytic domain illustrating three conserved salt-bridges. Residues participating in each salt-bridge are colored and labeled. The three inhibitors are shown as sticks.

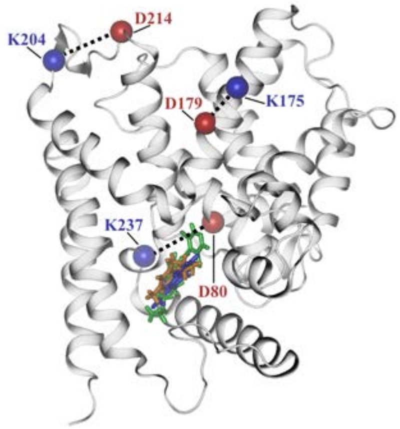

Supplement: S13 Fig — Residues participating in each salt-bridge are colored and labeled. The three inhibitors are shown as sticks. (PDF) [file pone.0214554.s017.pdf]
